# Single-Site Iridium Picolinamide Catalyst Immobilized onto Silica for the Hydrogenation of CO_2_ and the Dehydrogenation of Formic Acid

**DOI:** 10.1021/acs.inorgchem.2c01640

**Published:** 2022-06-29

**Authors:** Leonardo Tensi, Alexander V. Yakimov, Caterina Trotta, Chiara Domestici, Jordan De Jesus Silva, Scott R. Docherty, Cristiano Zuccaccia, Christophe Copéret, Alceo Macchioni

**Affiliations:** † Department of Chemistry, Biology and Biotechnology and CIRCC, Università degli Studi di Perugia, Perugia 06123, Italy; ‡ Department of Chemistry and Applied Biosciences, ETH Zurich, Zurich 8093, Switzerland

## Abstract

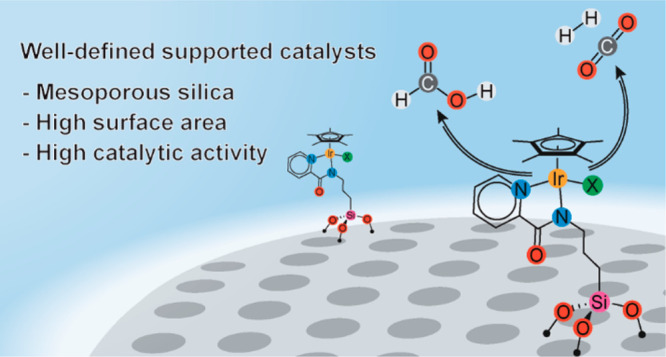

The development of an efficient heterogeneous catalyst for storing H_2_ into CO_2_ and releasing it from the produced formic acid, when needed, is a crucial target for overcoming some intrinsic criticalities of green hydrogen exploitation, such as high flammability, low density, and handling. Herein, we report an efficient heterogeneous catalyst for both reactions prepared by immobilizing a molecular iridium organometallic catalyst onto a high-surface mesoporous silica, through a sol–gel methodology. The presence of tailored single-metal catalytic sites, derived by a suitable choice of ligands with desired steric and electronic characteristics, in combination with optimized support features, makes the immobilized catalyst highly active. Furthermore, the information derived from multinuclear DNP-enhanced NMR spectroscopy, elemental analysis, and Ir L_3_-edge XAS indicates the formation of cationic iridium sites. It is quite remarkable to note that the immobilized catalyst shows essentially the same catalytic activity as its molecular analogue in the hydrogenation of CO_2_. In the reverse reaction of HCOOH dehydrogenation, it is approximately twice less active but has no induction period.

## Abstract 2

We report the synthesis of a heterogeneous immobilized catalyst (**Ir_PicaSi_SiO**_**2**_) and its successful application in aqueous CO_2_ hydrogenation and FA dehydrogenation. The information derived from multinuclear DNP-enhanced NMR spectroscopy, elemental analysis, and XAS indicates the presence of cationic iridium sites in **Ir_PicaSi_SiO**_**2**_. The latter shows essentially the same catalytic activity as its molecular analogue in the hydrogenation of CO_2_. In the reverse reaction of HCOOH dehydrogenation, it is approximately twice less active but has no induction period.

## Introduction

Energy transition from fossil to renewable fuels is the ultimate challenge of our society which might be successfully faced through the implementation of an efficient technology for producing “green” hydrogen, i.e., hydrogen derived from the photoelectrocatalytic splitting of water.^1−6^ H_2_ is surely a clean primary energy carrier having, nevertheless, with its high flammability, low density, and handling, some serious criticalities.^7,8^

An interesting alternative to using H_2_ as such is to store it in the so-called liquid/solid hydrogen carriers and regenerate it when needed. One of the most promising H_2_ carriers is formic acid (FA),^9−12^ which can be generated by storing H_2_ into CO_2_, from which H_2_ might be reformed by the reverse reaction.

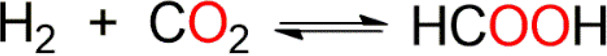



**Scheme 1:**
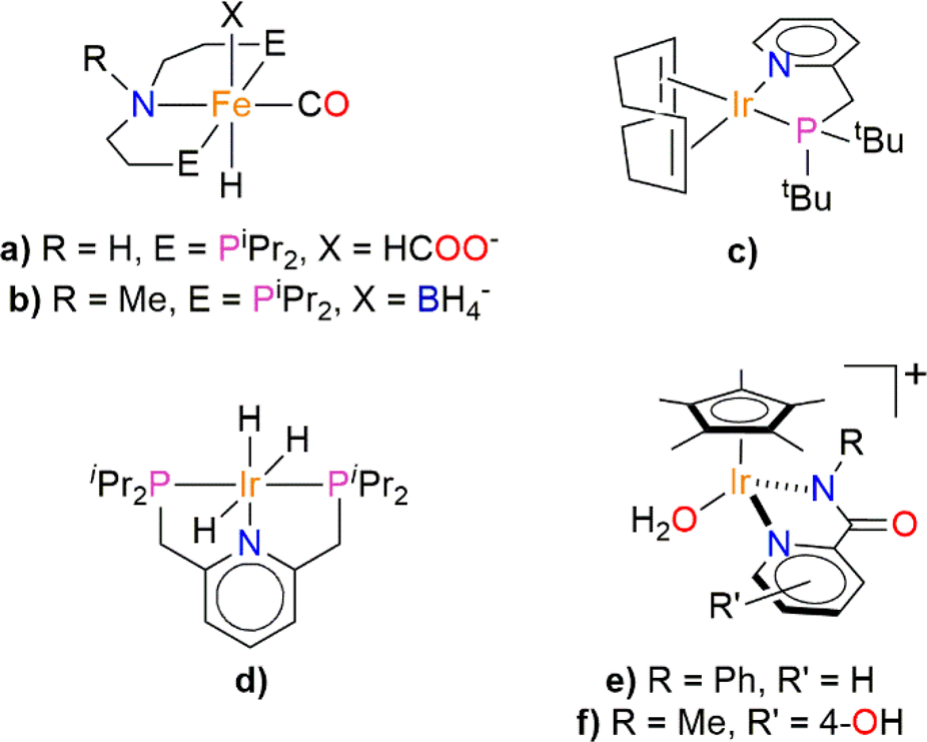
Relevant Organometallic Catalysts for FA Dehydrogenation and CO_2_ Hydrogenation

Both forward and back reactions ask for suitable catalysts in order to be viable, and several efficient catalysts have been reported so far.^9,10,12^ Supported nanoparticles of noble metals such as Pd and AuPd^13−17^ and, especially, organometallic complexes,^18−20^ mainly based on Fe,^21−31^ Ru,^32−40^ and Ir,^41−44^ have shown promising catalytic performances in terms of both turnover numbers (TONs) and turnover frequencies (TOFs). Notably, Hazari and Bernskoetter reported a class of iron complexes (a and b in Scheme 1) capable of reaching very high values of TON (ca. 10^6^ in FA dehydrogenation and 6 × 10^4^ in CO_2_ hydrogenation).^21,23,31^ Unfortunately, the conditions involve organic solvents and the presence of Lewis acids. Williams reported an Ir^I^-based complex (c in Scheme 1)^42^ with remarkable performance in FA dehydrogenation (TON value over 2 × 10^6^ with a maximum TOF of 3.7 s^–1^) carried out in neat FA, whereas Nozaki developed an Ir^III^ pincer trihydride catalyst (complex d in Scheme 1)^43,45^ very active in CO_2_ hydrogenation (TON = 3.5 × 10^6^ and TOF = 1.5 × 10^5^ h^–1^), operating in a basic aqueous solution, capable of catalyzing also FA dehydrogenation in *^t^*BuOH, in the presence of NEt_3_.

Cp*Ir-based (Cp* = pentamethylcyclopentadienyl) complexes have been successfully exploited as efficient catalysts for both CO_2_ hydrogenation and FA dehydrogenation in water, under mild conditions, without using any additive, except a base in the case of CO_2_ hydrogenation.^46−51^

Remarkable results have been obtained by Himeda, Fujita, and co-workers using [Cp*Ir(R-pica)X] (pica = picolinamide) complexes (e and f in Scheme 1)^52,53^ in both FA dehydrogenation^52,54,55^ and CO_2_ hydrogenation.^53,56−58^ Originally introduced by Watanabe and co-workers for the preparation of amine compounds,^59^ [Cp*Ir(R-pica)X] complexes are a successful class of compounds that have been found to efficiently catalyze many other reactions including transfer hydrogenation in cell growth media,^60,61^ reductive amination of ketones,^62−64^ water oxidation,^65^ NADH regeneration,^65,66^ hydrogenolysis of halosilanes,^67^ and hydrogen peroxide generation.^68^ They also exhibited good performance as antimicrobial, antibacterial, and anticancer agents.^69−72^

Considering the versatility and success of [Cp*Ir(R-pica)X] complexes as catalysts, we decided to immobilize them into mesoporous silica, aiming at preparing a hybrid single-site organometallic heterogeneous catalyst, having the distinctive features of the analogous molecular catalysts, adding all the advantages of heterogeneous catalysts in terms of recoverability and process intensification.^73−76^ Mesoporous silica was selected as support since it is inert and inexpensive, easily recoverable from the reaction mixtures, and characterized by a high surface area, thus facilitating the exposure of the catalytic species to the reactants and maximizing the exploitation of the noble metal.^77^

**Scheme 2:**
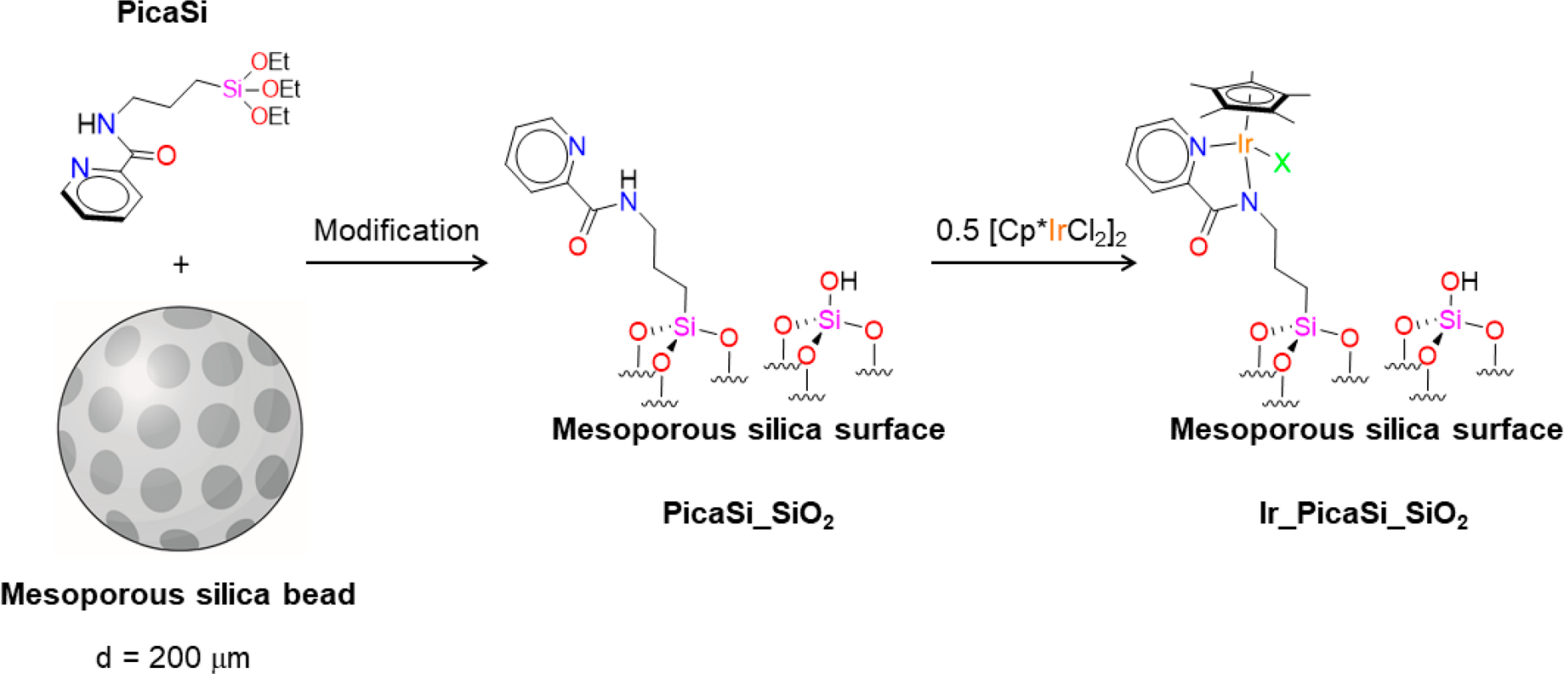
Steps of the Synthesis of **Ir_PicaSi_SiO**_**2**_

Herein, we report the synthesis of a heterogeneous immobilized catalyst (**Ir_PicaSi_SiO**_**2**_) and its successful application in CO_2_ hydrogenation and FA dehydrogenation. The preparation of **Ir_PicaSi_SiO**_**2**_ involved the initial synthesis of a modified version of the pica ligand (**PicaSi**), in which R is the (3-triethoxysilyl)propyl moiety, and the immobilization of **PicaSi** onto mesoporous silica (**PicaSi_SiO**_**2**_) via a sol–gel process, which was recently reported as an effective strategy to provide homogeneously distributed ligands on high surface area materials.^78−80^ The catalytic iridium-single site was then implanted by the reaction of **PicaSi_SiO**_**2**_ with [Cp*IrCl_2_]_2_ (**Ir_PicaSi_SiO**_**2**_) (Scheme 2).

To obtain a deeper molecular-level understanding of the surface, both materials were investigated via solid-state NMR spectroscopy and XAS. In order to increase the sensitivity of NMR toward surface sites, the dynamic nuclear polarization surface-enhanced NMR spectroscopy (DNP-SENS) approach was exploited. This technique allows the increase of NMR sensitivity by up to 2 orders of magnitude and thereby the recording of natural abundance ^13^C, ^15^N, and ^29^Si solid-state NMR spectra in a reasonable acquisition time.^81−85^**Ir_PicaSi_SiO**_**2**_ exhibited remarkable catalytic performances in aqueous FA dehydrogenation and CO_2_ hydrogenation that compare well with those of the analogous molecular catalysts. Extensive kinetic studies revealed that the reaction pathway for supported catalysts differs from the analogous molecular systems, because of the formation of cationic sites stabilized by the surface.

## Results and Discussion

We will first discuss the preparation and comprehensive characterization of **Ir_PicaSi_SiO**_**2**_ (Section 1) and then its application as a catalyst (Section 2). The first section provides evidence regarding the nature of the surface sites thanks to the use of state-of-the-art characterization, while the second section focuses on the performance of the catalyst and includes detailed catalytic and kinetic tests in both the CO_2_ hydrogenation and the reverse reaction, FA dehydrogenation.

### 1 Preparation and Characterization of **Ir_PicaSi_SiO**_**2**_

The hybrid material **Ir_PicaSi_SiO**_**2**_ was prepared by a two-step procedure involving 1a) the synthesis and immobilization onto mesoporous SiO_2_ of a pica-modified ligand (**PicaSi_SiO**_**2**_) via a sol–gel process and 1b) a postfunctionalization to incorporate the iridium organometallic moiety (**Ir_PicaSi_SiO**_**2**_), taking advantage of the coordination ability of **PicaSi_SiO**_**2**_ (Scheme 2).

#### 1a Synthesis and Heterogenization of **PicaSi** onto SiO_2_

A modified version of the picolinamide ligand (**PicaSi**, Scheme 2) having a (3-triethoxysilyl)propyl group on the nitrogen atom of the amide moiety was prepared, following reported procedures (SI).^86,87^**PicaSi** was successively immobilized via a sol–gel procedure onto commercially available mesoporous silica beads (**PicaSi_SiO**_**2**_), that are easy to handle and to recover due to a convenient particle diameter (from 60 to 200 μm). This support is also characterized by high surface area (738 m^2^ g^–1^), which is ideal for catalysis.^79,80^

Specifically, TEOS (tetraethyl orthosilicate), **PicaSi**, and silica beads were contacted in an acidified solution in THF, at 343 K for 1 h. The collected solid was washed with water/THF, ethanol, and diethyl ether and dried at 408 K under high vacuum. The thus-obtained material (**PicaSi_SiO**_**2**_) was characterized by means of IR spectroscopy, low-temperature N_2_ adsorption (BET), elemental analysis (EA), and DNP-SENS. In order to assign the NMR resonances, DFT calculations were also conducted.

The Diffuse Reflectance Infrared Fourier Transform (DRIFT) spectrum of **PicaSi_SiO**_**2**_ (Figure S1, SI) displays all the characteristic bands of the picolinamide moiety, namely the C=O stretching (1670 cm^–1^) of the amide and the C=C stretching (1545 cm^–1^) of the aromatic carbons of the pyridine ring as well as the classic aromatic (3065 cm^–1^) and aliphatic CH stretching (2964 and 2887 cm^–1^). EA confirms the presence of nitrogen and carbon on the solid (SI). The incorporation of the ligand onto the surface caused a reduction of the surface area from 738 m^2^ g^–1^ (starting mesoporous silica) to 650 m^2^ g^–1^ (**PicaSi_SiO**_**2**_).

**Figure 1:**
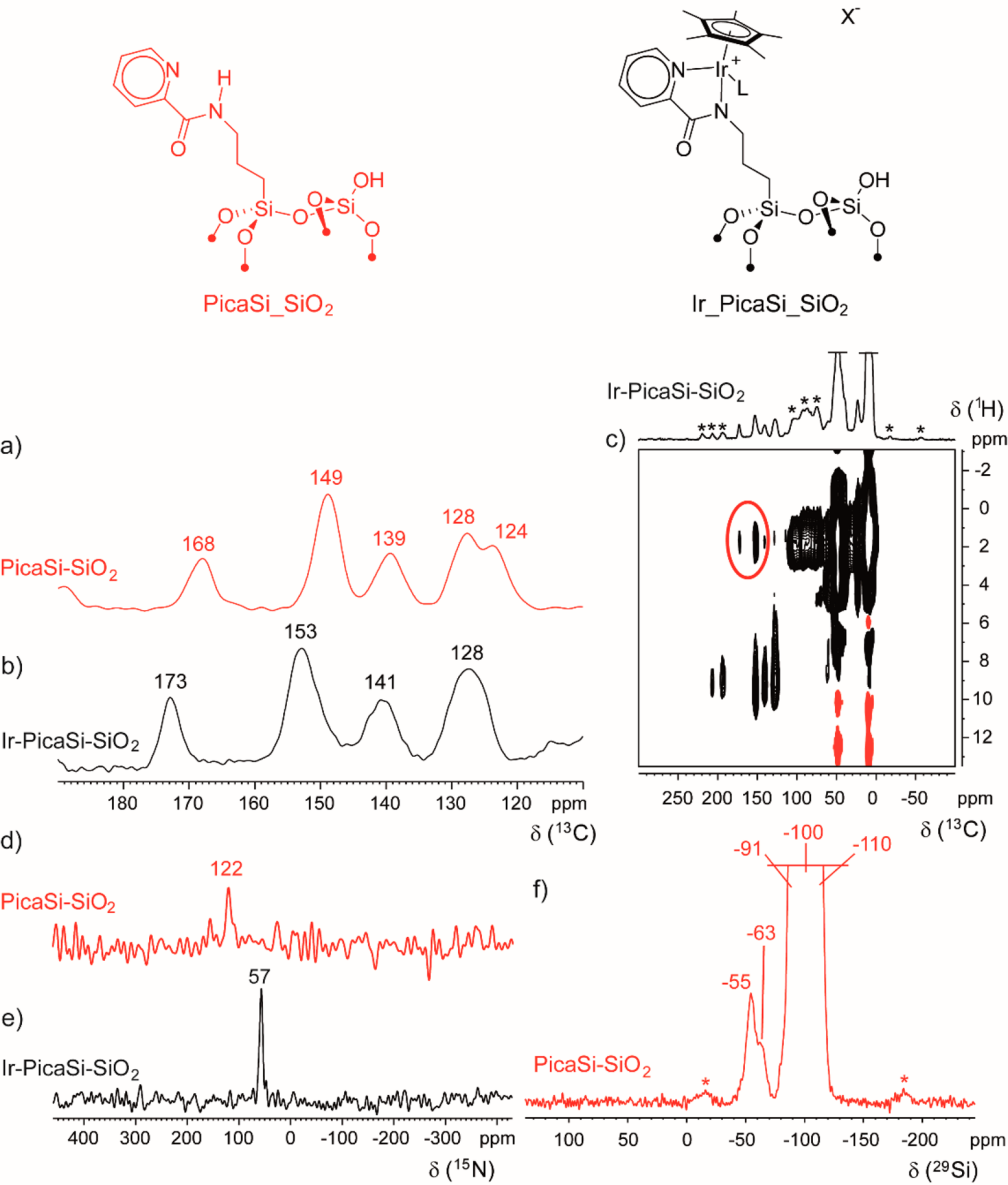
DNP-enhanced MAS NMR spectra of **PicaSi_SiO**_**2**_ and **Ir_PicaSi_SiO**_**2**_ materials. a) ^13^C CPMAS NMR spectrum of **PicaSi_SiO**_**2**_ (MAS 10 kHz, 100 K), b) ^13^C CPMAS NMR spectrum of **Ir_PicaSi_SiO**_**2**_ (MAS 10 kHz, 100 K), c) {^1^H}^13^C HETCOR NMR spectrum of **Ir_PicaSi_SiO**_**2**_ (MAS 10 kHz, 100 K, black = positive, red = negative), d) ^15^N CPMAS NMR spectrum of **PicaSi_SiO**_**2**_ (MAS 10 kHz, 100 K), e) ^15^N CPMAS NMR spectrum of **Ir_PicaSi_SiO**_**2**_ (MAS 10 kHz, 100 K), and f) ^29^Si CPMAS NMR spectrum of **PicaSi_SiO**_**2**_ (MAS 10 kHz, 100 K). Asterisks mark spinning sidebands.

In order to further understand the structure of the ligand on the surface, **PicaSi_SiO**_**2**_ was characterized via DNP-SENS. Using a typical sample formulation (**PicaSi_SiO**_**2**_ with an aqueous solution of AMUPol biradical to hyperpolarize the surface)^88,89^ and DNP-SENS acquisition conditions (100 K, constant microwave (MW) irradiation and cross-polarization DNP cross effect),^85^ we recorded spectra with DNP solvent enhancement of the NMR sensitivity reaching 120. The DNP-enhanced ^13^C cross-polarization magic angle spinning (CPMAS) NMR spectrum exhibits (i) three different aliphatic resonances (at 43, 23, and 9 ppm) that can be assigned to the three CH_2_ of the propyl moiety that bonds the ligand onto the surface, (ii) five resonances in the aromatic region, and (iii) a resonance centered at 168 ppm consistent with the quaternary carbon of the amide moiety (Figure 1a). Interestingly, the pattern of resonances of the ^13^C NMR spectrum of **PicaSi_SiO**_**2**_ matches that of the **PicaSi** ligand in CD_2_Cl_2_ solution (Figure S3), indicating structural similarities between the immobilized and molecular organic moieties.

To determine the binding mode of the ligand to the surface, the ^29^Si DNP-SENS NMR spectrum of **PicaSi_SiO**_**2**_ was also recorded. In addition to the classical resonances of the amorphous silica at ca. −90 to −110 ppm, two peaks centered at −55 and −63 ppm are observed (Figure 1f), showing that the ligand is bound to the surface of the material predominantly in T2 and T3 fashion.^90^ This result is in agreement with the findings of DFT computational studies conducted for various binding modes of the ligand to the SiO_2_ surface (summarized in Table S2; detailed descriptions are in the SI).

To further understand the structure of the immobilized ligand, ^15^N NMR spectroscopy was also carried out. DNP-enhanced spectroscopy was used in order to enable such studies at ^15^N natural abundance. The spectrum (Figure 1d) shows a resonance at 122 ppm assigned to the amidic nitrogen, while the resonance of the pyridinic nitrogen was not detected. The absence of the latter is likely due to the larger chemical shift anisotropy of the signal that decreases sensitivity.^91^

Overall, all pieces of information confirm that **PicaSi** is present on the surface of the mesoporous SiO_2_, and particularly, DFT calculations in combination with ^29^Si DNP-SENS allow establishing that the ligand is linked to the silica surface through the silane moiety predominantly in T2 and T3 fashion.

#### 1b Immobilization of an Iridium Organometallic Moiety

The immobilization of Ir was next carried out by reacting a solution of [Cp*IrCl_2_]_2_ with **PicaSi_SiO**_**2**_ in anhydrous dichloromethane, under inert atmosphere, for 48 h at RT, in the presence of a base (NEt_3_) (SI). The resulting material **Ir_PicaSi_SiO**_**2**_ shows a small decrease of surface area from 650 m^2^ g^–1^ (**PicaSi_SiO**_**2**_) to 595 m^2^ g^–1^ (**Ir_PicaSi_SiO**_**2**_). The C:N:Ir:Cl experimental ratio in **Ir_PicaSi_SiO**_**2**_ (19.4:2.0:1.0:0.3), obtained by EA, is close to the expected one (19.0:2.0:1.0:1.0), albeit with a slight chlorine deficiency that could be due to a partial replacement of Cl^–^ by OH^–^ or HSO_4_^–^ (SI, page S7). Based on the EA and BET measurements, the surface density of Ir is ≈1 per 10 nm^2^. This result suggests that organometallic sites are well separated from each other, decreasing the probability of having multimetallic processes during catalytic reactions.

The DRIFT spectrum of **Ir_PicaSi_SiO**_**2**_ (Figure S4, SI) shows all the expected bands of the organic moiety on the surface with appreciable shifts with respect to the spectrum of **PicaSi_SiO**_**2**_ consistent with the binding of iridium on the immobilized ligand. In particular, C=O and C=C stretchings shift from 1670 to 1625 cm^–1^ and from 1545 to 1595 cm^–1^, respectively.

The DNP-enhanced ^13^C NMR spectrum shows all resonances of the Cp* and pica moieties confirming the presence of the iridium complex onto the material (Figure 1b). Interestingly, the resonance belonging to the quaternary carbon of the amide moiety shifts at an appreciably higher frequency, from 168 to 173 ppm, consistent with the coordination of the metal center to the immobilized organic ligand. The dipolar coupling between the resonances of pica and Cp*, observed in the DNP-enhanced 2D {^1^H}^13^C HETCOR NMR spectrum, further confirms the spatial proximity of the two moieties (Figure 1c). Additionally, the ^15^N chemical shift of the amide moiety decreased from 122 to 57 ppm (Figure 1e), pointing to the coordination of the ligand to iridium.

**Figure 2:**
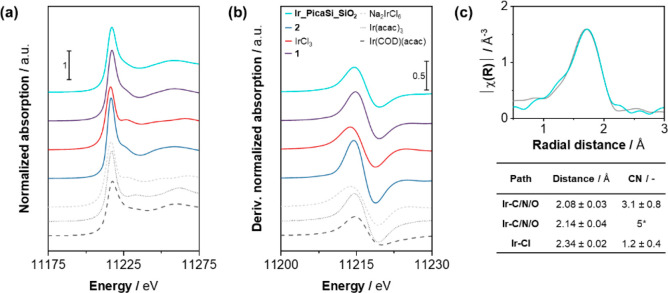
XAS data of **Ir_PicaSi_SiO**_**2**_ and selected reference compounds. a) Normalized Ir L_3_ edge XANES and b) the first derivative of Ir L_3_ edge XANES. From bottom to top: Ir(COD)(acac) (dark gray, dash), Ir(acac)_3_ (gray, dot), Na_2_IrCl_6_ (light gray, short dash), **2** (dark blue), IrCl_3_ (red), **1** (purple), and **Ir_PicaSi_SiO**_**2**_ (turquoise). c) k^2^-weighted R-space EXAFS data (turquoise) and fitting (gray) with the corresponding fitting parameters summarized in the table (for full details of fit see Table S5, SI).

In order to gain further information about the local surrounding of Ir in **Ir_PicaSi_SiO**_**2**_, X-ray absorption spectroscopy (XAS) studies were performed at the iridium L_3_-edge. X-ray absorption near-edge structure (XANES, Figure 2) edge energies of **Ir_PicaSi_SiO**_**2**_ and the molecular analog [(Cp*)Ir(*N*-propyl-pica)Cl] (**1**) (**1** is a new complex, and its synthesis and characterization are reported in the SI from page S11 to page S13) are identical and equal to 11214.75 eV (Table S3, entries 1 and 2) and very close to that of Ir(acac)_3_ (acac = acetylacetonate) and [Cp*IrCl_2_]_2_ (**2**) (11214.75 and 11214.5 eV, respectively). Notably, for IrCl_3_, the edge energy (11213.75 eV) is shifted to a lower value than the other references found in the +3 oxidation state, likely as a result of the decreased covalency of the Ir–Cl bonds. Further comparison of the edge energy to reference compounds in the +1 (Ir(cod)(acac), 11215.0 eV, cod = 1,5-cyclooctadiene) and +4 (Na_2_IrCl_6_, 11213.75 eV) oxidation states suggests that the average oxidation state is most likely +3 for the supported system. Comparison of the white line intensity for the series of compounds measured indicates that, as expected, a more intense white line feature corresponds to higher average oxidation states (Table S3), while **2** possesses an anomalously high white line intensity (2.80).

Subsequently, analysis of the extended X-ray absorption fine structure (EXAFS) was performed to ascertain the identity of the nearest neighbors of Ir in the supported system (**Ir_PicaSi_SiO**_**2**_). It is noteworthy that EXAFS provides information related to the average coordination of Ir in the material, rather than a specific coordination geometry, or indeed a single, unified coordination environment.^92,93^

Fitting of the k^2^-weighted EXAFS for the supported catalyst **Ir_PicaSi_SiO**_**2**_ is consistent with a material containing Ir sites with C/N/O scattering paths (8.1 ± 0.8) and Cl scattering paths (1.2 ± 0.4) (Figure S14, parameters summarized in Table S5). Since the light atoms (C/N/O) are similar in mass, they cannot be easily distinguished by EXAFS.^94,95^ As such, the C/N/O scattering paths are separated into two groups for fitting–those arising from the Cp* ligand, which have constrained path degeneracy (*N* = 5, *R* = 2.14 ± 0.04 Å), and those assigned to the picolinamide moiety and C,N,O-based ligands (H_2_O, SO_4_^2–^) (*N* = 3.1 ± 0.8, *R* = 2.08 ± 0.03 Å), giving a total C/N/O coordination of (8.1 ± 0.8). To obtain a reasonable fit, it was necessary to also include an Ir–Cl path, the degeneracy of which was found to be (*N* = 1.2 ± 0.4, *R* = 2.34 ± 0.02 Å) from the fit obtained. The presence of an Ir–Cl path is consistent with the data from elemental analysis, which suggests that some chloride is retained after synthesis.

For **Ir_PicaSi_SiO**_**2**_, the combination of elemental analysis and the assignment made on the basis of the obtained EXAFS fits suggests that there are two distinct Ir species present in the material, both of which contain both Cp* and picolinamide moieties bound to Ir, as well as an additional ligand which can be either a chloride anion or a water molecule.

In order to address this issue further, reaction of **Ir_PicaSi_SiO**_**2**_ with ^15^*N*-pyridine (Py) was carried out, and the recovered material was studied by means of DNP-enhanced ^15^N CPMAS NMR (Figure S13, SI).^96^ Two resonances were observed at 219 and 287 ppm corresponding to the interaction of Py with iridium and silanol groups, respectively. Whereas the latter one is commonly observed in silica-supported materials,^97^ the presence of the former interaction indeed further suggests that iridium sites in **Ir_PicaSi_SiO**_**2**_ have a cationic character.^98^

The general picture emerging from such an in-depth characterization indicates that **Ir_PicaSi_SiO**_**2**_ is a hybrid material in which rather dispersed cationic iridium sites are coordinated at **PicaSi** moieties anchored onto the silica surface.

### 2 Catalytic Applications of **Ir_PicaSi_SiO**_**2**_

#### 2a FA Dehydrogenation

**Ir_PicaSi_SiO**_**2**_ was tested as a catalyst in the FA dehydrogenation to CO_2_ and H_2_ at different pH values (1.4–8.2 range, entries 1–5, Table 1), catalyst concentrations (25–500 μM range, entries 6–11, Table 1), [HCOOH]+[HCOO^–^] concentrations (0.5–5 M range, entries 3, 9, and 12–14, Table 1), and temperatures (288–353 K range, entries 7 and 15–18, Table 1). The progress of the reactions was followed by means of differential manometry and solution NMR spectroscopy. The experiments were conducted by adding formic acid to a suspension of **Ir_PicaSi_SiO**_**2**_ in a HCOO^–^ solution. Conversion was evaluated by measuring the amount of residual formic acid/formate via ^1^H NMR spectroscopy. The complex [Cp*Ir(N-Me-pica)Cl] (**3**) was used as a literature benchmark.^54,55^ All the results are summarized in Table 1.

**Table 1:** Summary of the Performances of **Ir_PicaSi_SiO**_**2**_ in the FA Dehydrogenation Reaction

	[Cat] (μM)	[HCOOH]+[HCOO^–^] (M)	[HCOOH] (M)	pH	*T* (K)	TOF (h^–1^)	TON	convn (%)
**1**	250	3	3	1.4	298	254	12000	>99
**2**	250	3	2.8	2.4	298	480	11200	>99
**3**	250	3	1.5	3.7	298	636	5640	94
**4**	250	3	0.8	4.2	298	505	3370	>99
**5**	250	3	0	8.2	298	2	120	
**6**	25	1	0.5	3.7	298	425	17600	88
**7**	50	1	0.5	3.7	298	448	9200	92
**8**	100	1	0.5	3.7	298	354	5100	>99
**9**	250	1	0.5	3.7	298	469	2160	>99
**10**	350	1	0.5	3.7	298	450	1490	>99
**11**	500	1	0.5	3.7	298	359	1020	>99
**12**	250	0.5	0.25	3.7	298	212	1000	>99
**13**	250	0.75	0.375	3.7	298	235	1410	94
**14**	250	5	2.5	3.7	298	890	10400	>99
**15**	50	1	0.5	3.7	288	105	5600	56
**16**	50	1	0.5	3.7	313	1070	7200	72
**17**	50	1	0.5	3.7	333	5400	7400	74
**18**	50	1	0.5	3.7	353	11200	5800	58

**Figure 3:**
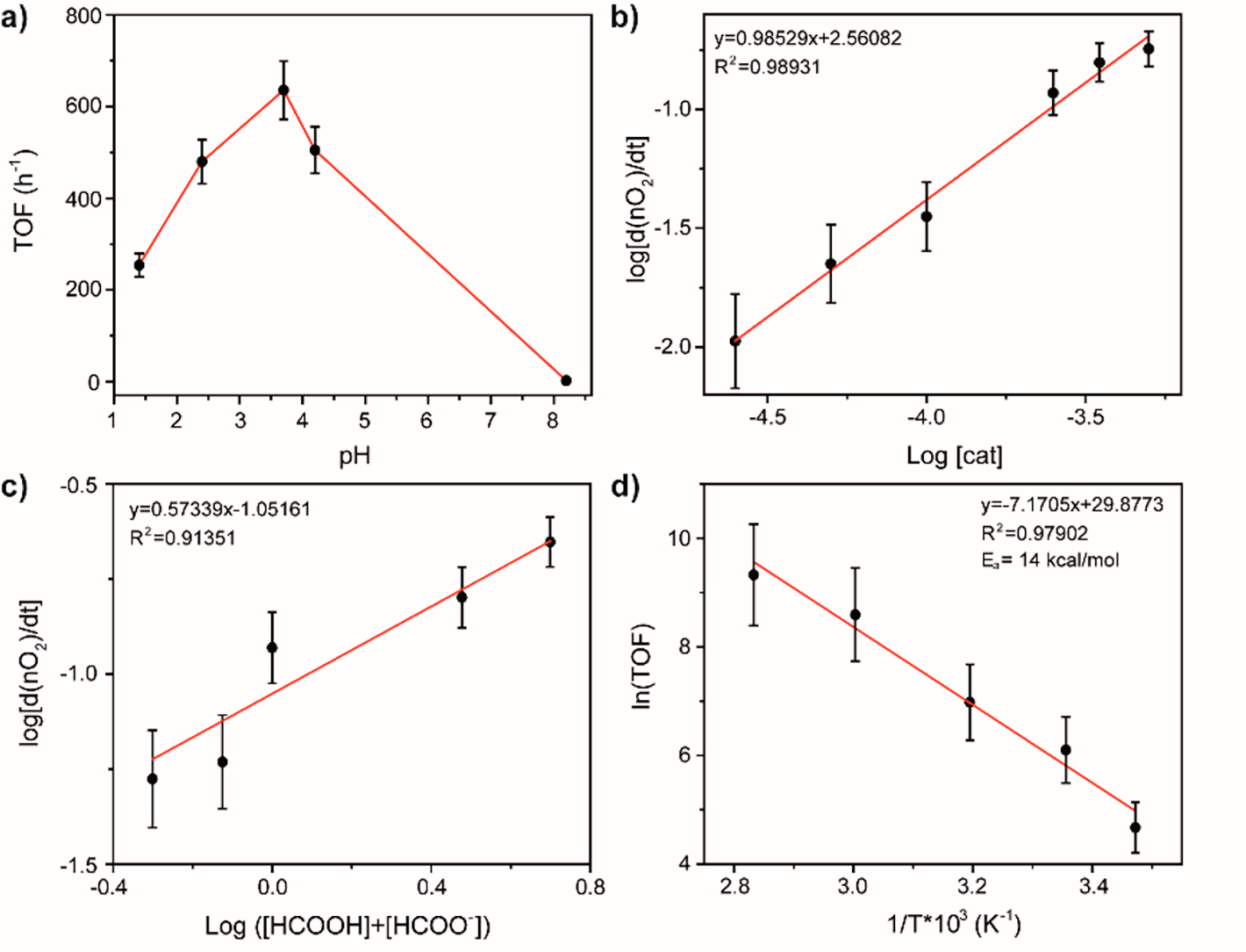
Kinetic trends of the performances of **Ir_PicaSi_SiO**_**2**_ in the FA dehydrogenation reaction. a) TOF (h^–1^) vs pH trend for formic acid dehydrogenation catalyzed by **Ir_PicaSi_SiO**_**2**_ ([cat] = 250 μM, [HCOOH]+[HCOO^–^] = 3 M, 298 K). b) log(d(*n*O_2_)/d*t*) vs log[cat] for formic acid dehydrogenation catalyzed by **Ir_PicaSi_SiO**_**2**_ ([HCOOH]+[HCOO^–^] = 1 M, pH = 3.7, 298 K). c) log[d(*n*O_2_)/d*t*] vs log([HCOOH]+[HCOO^–^]) for formic acid dehydrogenation catalyzed by **Ir_PicaSi_SiO**_**2**_ ([cat] = 250 μM, pH = 3.7, 298 K). d) ln(TOF) vs 1/*T* for formic acid dehydrogenation catalyzed by **Ir_PicaSi_SiO**_**2**_ in a temperature range 288–353 K ([cat] = 50 μM, [HCOOH]+[HCOO^–^] = 1 M, pH = 3.7).

**Scheme 3:**
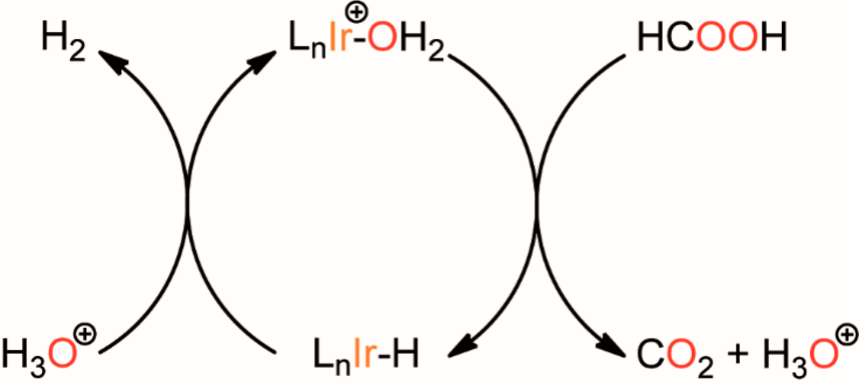
Simplified Reaction Mechanism of FA Dehydrogenation Mediated by Ir-Based Catalysts L = ancillary ligand.

TOF vs pH exhibits a volcano-shaped trend (entries 1–5, Table 1, Figure 3a). The highest TOF value (636 h^–1^) was observed around pH = 3.7, corresponding to the p*K*_a_ of the formic acid, as already reported for other Ir-based catalysts.^54,55^ This can be explained considering the simplified reaction mechanism illustrated in Scheme 3 involving two steps. The first step is the activation of the C–H bond of FA, leading to a L_*n*_Ir–H intermediate, generating CO_2_ and H_3_O^+^; in the second step, the protonation of the L_*n*_Ir–H species liberates H_2_. Indeed, low pH makes the deprotonation of HCOOH and the consequent formation of Ir–H complicated, whereas at higher pH, the protonation of Ir–H and the consequent evolution of H_2_ become slightly probable.^99^

TOF values were found to be slightly dependent on the concentration of **Ir_PicaSi_SiO**_**2**_ (354–469 h^–1^, entries 6–11, Table 1, Figure 3b), indicating a first-order dependence on catalyst concentration. On the other hand, the trend of TOF vs [HCOOH]+[HCOO^–^] (entries 3, 9, and 12–14, Table 1, Figure 3c) suggests a noninteger reaction order (0.6) on FA concentrations. In all cases, quantitative consumption of HCOOH was achieved.

Catalytic tests at different temperatures (288–353 K, entries 7 and 15–18, Table 1) show an increase of TOF from 105 h^–1^ at 288 K to 11200 h^–1^ at 353 K. The apparent activation energy (*E*_a_ = 14 ± 1 kcal mol^–1^), evaluated from the Arrhenius plot (Figure 3d), is considerably lower than that of the previously determined value for **3** (*E*_a_ = 20 kcal mol^–1^).^54^ This can be explained considering an active role of the Si–OH functionalities, which might facilitate the deprotonation of FA and/or the protonation of Ir–H, under the assumption that the thermodynamics of HCOOH adsorption on the surface is not affecting the apparent activation energy.

**Figure 4:**
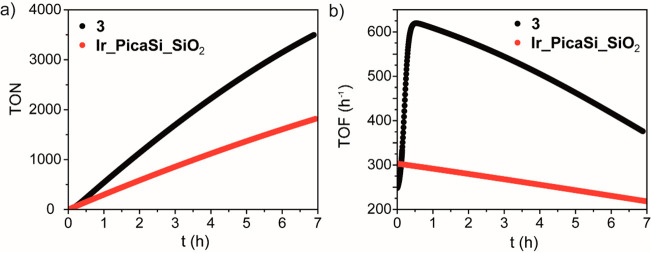
Kinetic trends of the performances of **Ir_PicaSi_SiO**_**2**_ and **3** in the FA dehydrogenation reaction. a) TON vs *t* (h) ([HCOOH]+[HCOO^–^] = 1 M, pH = 3.7, [Cat] = 25 μM, *T* = 298 K) for **3** and **Ir_PicaSi_SiO**_**2**_. b) TOF (h^–1^) vs *t* (h) ([HCOOH]+[HCOO^–^] = 1 M, pH = 3.7, [Cat] = 25 μM, *T* = 298 K) for **3** and **Ir_PicaSi_SiO**_**2**_.

The activity and stability of the heterogenized catalyst **Ir_PicaSi_SiO**_**2**_ were compared to that of **3** by performing catalytic experiments under the same conditions ([Ir] = 25 μM, an equimolar concentration of [HCOOH] and [HCOO^–^] amounting at 1 M, pH = 3.7 and 298 K, TON_expected_ = 20000). Gas evolution was monitored by differential manometry for 7 h (Figure 4a). TON vs time trends were interpolated using a composite mathematical function developed by Peters and Baskin.^100^ The first derivative of these “smooth” trends allows obtaining the evolution of TOF versus time (Figure 4b).

Interestingly, albeit TOF_max_ of **Ir_PicaSi_SiO**_**2**_ (303 h^–1^) is ca. two times slower than that of **3** (620 h^–1^), at *t* = 0, the former exhibits a higher TOF (303 h^–1^) with respect to **3** (248 h^–1^) (Figure 4b). The trend of TOF versus time of **3** clearly shows an induction period of ca. 30 min, which is absent in that of **Ir_PicaSi_SiO**_**2**_ (Figure 4b). This induction period might be due to the possible formation of poorly active (or inactive), out-of-cycle, dinuclear species.^101^ As discussed above, most of the Ir sites in **Ir_PicaSi_SiO**_**2**_ are well separated (≈1 Ir every 10 nm^2^), making the associative process unlikely. After 6 h, the activity of **Ir_PicaSi_SiO**_**2**_ decreased to 231 h^–1^, whereas that of **3** decreased down to 418 h^–1^. This decrease of activity is more accentuated than that expected based on the 0.4 and 0.6 dependence of the reaction rate on the concentration of formic acid in homogeneous and heterogeneous catalysis, respectively, and of similar entities, suggesting that catalyst transformation/deactivation processes occur for both catalysts. The main degradative pathway of complex **3** is the reductive deoxygenation of the C=O moiety of the ligand to form the corresponding amino species;^55^ the same degradation mechanism might also take place in the case of **Ir_PicaSi_SiO**_**2**_. Nevertheless, after 7 days, both **Ir_PicaSi_SiO**_**2**_ and **3** reached a TON of 17600, over the 20000 expected cycles based on thermodynamics, corresponding to 88% of conversion.

The recoverability of **Ir_PicaSi_SiO**_**2**_ was evaluated by performing successive tests in which the catalyst was separated and reused in the dehydrogenation of fresh FA aqueous solutions ([HCOOH]+[HCOO^–^] = 1 M, pH = 3.7, 298 K) (see the SI for experimental details). After each catalytic run, Ir leaching from the solid was determined by means of Inductively Coupled Plasma-Atomic Emission Spectroscopy (ICP-AES) analysis, and the activity of the separated supernatant solution was tested. The activities of **Ir_PicaSi_SiO**_**2**_ and those of the respective supernatant solutions along with Ir content and leaching for each run are reported in Table S6.

The percentage of Ir leaching in each run, as detected by ICP-AES analysis, is quite low (Ir concentration in the material passed from 0.115 μmol/mg to 0.100 μmol/mg after four cycles, Table S6). The recovered supernatant solution after each run was not active. On the other hand, **Ir_PicaSi_SiO**_**2**_ can be reused successfully for four successive runs, albeit a progressive decrease (from 385 h^–1^ to 34 h^–1^) of TOF is observed (Table S6).

Overall, the supported catalyst **Ir_PicaSi_SiO**_**2**_ showed remarkable performance in FA dehydrogenation comparable to those of its homogeneous analogue **3** and of the best heterogenized iridium catalysts reported so far. Particularly, **Ir_PicaSi_SiO**_**2**_ reached a TOF value up to 11200 h^–1^ ([HCOOH]+[HCOO^–^] = 1 M, pH = 3.7, [**Ir_PicaSi_SiO**_**2**_] = 250 μM, *T* = 353 K) and a TON value up to 17600 ([HCOOH]+[HCOO^–^] = 1 M, pH = 3.7, [**Ir_PicaSi_SiO**_**2**_] = 25 μM, *T* = 298 K) in aqueous solution and in the absence of any additives. The catalyst was reused four times, and ICP-AES analysis of the supernatant solutions showed a low Ir leaching for each run. However, from the kinetic data, it is possible to observe a decrease of activity over time consistent with the presence of active catalyst transformation/deactivation processes.^55^

#### 2b CO_2_ Hydrogenation

**Ir_PicaSi_SiO**_**2**_ was also tested as a catalyst for the selective CO_2_ hydrogenation to formate under batch conditions. Typically, the reaction was carried out for 24 h at 423 K and 50 atm (CO_2_:H_2_ = 1:1) in the aqueous solution, in the presence of an organic base. The amount of produced formate was quantified by ^1^H NMR spectroscopy using 3-trimethylsilylpropanesulfonate sodium salt as the standard; in all the experiments, no other product was observed. Complex **1** was used as the molecular benchmark of the reaction. In the absence of the catalysts, no appreciable formation of formate was observed. The catalytic performances of **Ir_PicaSi_SiO**_**2**_ were evaluated at different catalyst loadings (0.057–1.16 μmol range, entries 1–7, Table 2) and in the presence of different bases (entry 2, Table 2, and entries S1 and S2, Table S7).

**Table 2:** Summary of the Performances of **Ir_PicaSi_SiO**_**2**_ and **1** in the CO_2_ Hydrogenation Reaction

	cat.	Ir content (μmol)	*n*_Formate_ (mmol)	[Formate] (M)	base	*t* (h)	TON
**1**	**Ir_PicaSi_SiO**_**2**_	0.057	0.4	0.080	DABCO	24	6983
**2**	**Ir_PicaSi_SiO**_**2**_	0.115	0.67	0.134	DABCO	24	5836
**3**	**Ir_PicaSi_SiO**_**2**_	0.230	0.88	0.176	DABCO	24	3829
**4**	**Ir_PicaSi_SiO**_**2**_	0.461	1.07	0.215	DABCO	24	2327
**5**	**Ir_PicaSi_SiO**_**2**_	0.693	1.19	0.238	DABCO	24	1720
**6**	**Ir_PicaSi_SiO**_**2**_	0.925	1.16	0.232	DABCO	24	1253
**7**	**Ir_PicaSi_SiO**_**2**_	1.16	1.36	0.273	DABCO	24	1178
**8**	**1**	0.120	0.63	0.126	DABCO	24	5235

**Figure 5:**
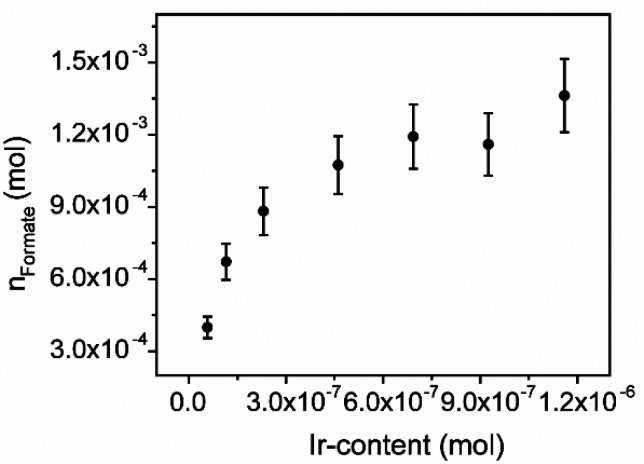
Trend of the *n*_Formate_ vs **Ir_PicaSi_SiO**_**2**_ content. Experimental conditions: *t* = 24 h, *T* = 423 K, *P* = 50 atm with CO_2_:H_2_ = 1:1 ratio, [DABCO] = 1 M.

**Ir_PicaSi_SiO**_**2**_ showed comparable performance to its molecular counterpart **1** (entries 2 and 8, Table 2). The effect of catalyst loading on the catalytic performances was explored performing the reaction in 1 M 1,4-diazabicyclo[2.2.2]octane (DABCO) aqueous solutions (entries 1–7, Table 2, Figure 5). An increase of formate production was observed up to ca. 4.5 × 10^–7^ mol of Ir content (Figure 5); after that, a *plateau* is reached indicative of an equilibration between reagents and products, which precluded the possibility of determining a kinetic order on catalyst (Figure 5). However, the TON observed with the lowest Ir loading (TON = 7 × 10^3^, entry 1, Table 2) is comparable to that of other already reported supported Ir complexes under similar experimental conditions.^48,49,79,80^

In summary, **Ir_PicaSi_SiO**_**2**_ is an active catalyst for the selective hydrogenation of CO_2_ to formate with catalytic performance comparable to those of the molecular complex **1** and to those of the best heterogenized iridium catalysts reported so far.

## Conclusions

A hybrid catalyst consisting of the [Cp*Ir(R-pica)X] complex immobilized onto mesoporous silica (**Ir_PicaSi_SiO**_**2**_) was prepared by means of a sol–gel procedure and characterized by a battery of instrumental techniques. The latter allowed the understanding that **Ir_PicaSi_SiO**_**2**_ maintains a very high surface area (595 m^2^ g^–1^) and has a rather dispersed single-site Ir(III) center (≈1 Ir every 10 nm^2^), still bearing Cp*- and pica-ligands but with a coordination vacancy, generated by the substitution of a chloride ligand by a water molecule or a Si–OH moiety. These features are essential in determining the remarkable catalytic performance of **Ir_PicaSi_SiO**_**2**_ in both CO_2_ hydrogenation and FA dehydrogenation. CO_2_ is hydrogenated to formate with comparable performance to its molecular counterpart, under the same conditions (Table 2). Interestingly, a strict comparison of the catalytic performance of **Ir_PicaSi_SiO**_**2**_ and its molecular analogue in FA dehydrogenation shows that, whereas the former has a TOF about 2 times lower, the latter does not exhibit any induction time. This might be due to having Ir species in its cationic form or, most likely, by the inhibition of any associative deactivation process in **Ir_PicaSi_SiO**_**2**_.

Kinetic studies (effect of pH, catalyst and FA concentration, temperature) further reveal a strict analogy between **Ir_PicaSi_SiO**_**2**_ and its molecular counterpart also in terms of the reaction mechanism, strongly suggesting that the species involved in the catalytic cycle are the same. The parallelism appears to be also applicable to the degradation of the catalytic center that occurs through the reductive deoxygenation of the C=O moiety of the ligand to form the corresponding amino species in **Ir_PicaSi_SiO**_**2**_, as previously observed for analogous molecular catalysts.^55^ Having observed that such a degradation pathway is active also in the immobilized catalyst allowed the understanding that it occurs intramolecularly, indicating that only an inhibition of the amide moiety rotation might avoid it.^49^ Minimizing catalyst degradation might pave the way to the development of catalysts with TON large enough to be really applied in storing green hydrogen into CO_2_ and releasing it from FA when needed.

## Supporting Information Available

Details on materials and methods; synthesis and characterization of **PicaSi_SiO**_**2**_, **Ir_PicaSi_SiO**_**2**_, (*N*-propyl)picolinamide, and **1**; additional NMR spectra; XAS details; and additional catalytic data (PDF)
