# Supplementary material for: Single-Site Iridium Picolinamide Catalyst Immobilized onto Silica for the Hydrogenation of CO2 and the Dehydrogenation of Formic Acid
Source: Inorg Chem. 2022 Jun 29;61(27):10575–86. doi: 10.1021/acs.inorgchem.2c01640 (PMC9348825; doi:10.1021/acs.inorgchem.2c01640)
Supplement: Supplementary file 1 — ic2c01640_si_001.pdf [file ic2c01640_si_001.pdf]

Supporting Information

Single-Site Iridium Picolinamide Catalyst Immobilized onto Silica for the Hydrogenation of CO_2 and the Dehydrogenation of Formic Acid

Leonardo Tensi,[1,2] Alexander V. Yakimov,[2] Caterina Trotta,[1] Chiara Domestici,[1] Jordan de Jesus Silva,[2] Scott R. Docherty,[2] Cristiano Zuccaccia,[1] Christophe Copéret,[2,*] and Alceo Macchioni,[1,*]

[1] Dipartimento di Chimica, Biologia e Biotecnologie and CIRCC, Università degli Studi di Perugia, Via Elce di Sotto, 8-06123 Perugia, Italy.
[2] Department of Chemistry and Applied Biosciences, ETH of Zurich, Vladimir Prelog Weg 1–5, CH-8093, Zurich, Switzerland

Table of Contents

Materials and methods S2

Synthetic Details S5

DFT ^{29}Si NMR data S14

XAS Data S15

^{15}N SS MAS NMR spectrum of impregnated Ir_PicaSI_SiO$_2$ with ^{15}N-pyridine S18

Kinetic Data of FA dehydrogenation reactions S19

Kinetic Data of CO_2 hydrogenation reactions S22

References S23

Materials and Methods

Anhydrous THF and CH_2Cl_2 were purchased from Acros Organics B.V.B.A.. Anhydrous triethylamine from Fluorochem Ltd and silica gel (SiliaSphere PC, 60 Å, 70-230 mesh) were purchased from Fluorochem Ltd and SiliCycle Inc. All the other reagents and the organic solvents were purchased from Merck KGAA and used as received. Water was purified using a Milli-QUltrapure water purification system. $[Cp^*IrCl_2]_2$ (**2**),[1] complex [Cp*Ir(N-Me-picolinamide)Cl] (**3**)[2] and N-(3-(triethoxysilyl)propyl)picolinamide (**PicaSi**)[3,4] were synthesized following reported procedure.

IR spectroscopy

IR spectroscopy measurements were carried out on a Bruker Alpha FT-IR spectrometer equipped with the DRIFT transmission module QuickSnap™ accessory. Bands are reported in cm^{-1} from 1600 to 4000 cm^{-1} (lower wavenumbers become difficult to analyze due to silica saturation) and characterized as broad (br), strong (s), medium (m), or weak (w) relative to other bands from 1600 to 4000 cm^{-1}. Data were processed using the Bruker OPUS software package.

Low-temperature N_2 Adsorption measurements

Nitrogen Adsorption-Desorption measurements were carried out at 77 K using a BELSORB-Mini from BEL-JAPAN. Before N_2 adsorption, the samples were degassed at less than 10^{-4} Torr at 298 K for at least 5 hours. Both the pore volume and the peak pore diameter were calculated using the Barrett–Joyner–Halenda (BJH) method. The specific surface area (S_{BET}) was calculated according to the Brunauer–Emmett–Teller (BET) equation. For the data processing BEL-Master software package was used.

Elemental Analysis

Elemental analysis for Ir and Cl was performed by Mikroanalytisches Labor Pascher (Remagen, Germany), for C,N and H – by micro-laboratory of the Microlabor Service of ETH Zurich.

Solution NMR spectroscopy

Solution ^{1}H and ^{13}C NMR spectra were measured with the Bruker Avance III 200 MHz (4.7 T) and Bruker Avance III 400 MHz (9.4 T) spectrometers. ^{1}H and ^{13}C chemical shifts were referenced to the residual solvent signals. Data are reported as follows: chemical shift, multiplicity (s = singlet, d = doublet, ddd = doublet of doublets of doublets, t = triplet, td = triplet of doublets, q = quartet, se = sextet, m = multiplet), J-coupling constants (Hz). Bruker Topspin 3.2 software was used for the data processing.

DNP-SENS

The DNP-SENS experiments were performed on a Bruker 600 MHz (14.1 T) spectrometer by using a 3.2 mm HX or HXY probe. A gyrotron generated microwaves with a power of approximately 6 W at 395 GHz to drive the DNP cross effect. All the samples were prepared as follows: 25 mg of the material were impregnated with 30 µL of AmuPol radical 10 mM solution in 10%/90% H_2O/D_2O; the mixture was then quickly packed in a sapphire rotor with zirconia cap. The sapphire rotors were used for optimal microwave penetration. Rotors were inserted in the cryogenic probe within a short period of time (~5 min) and cooled to 100 K by a cryogenic heat exchanger system. In all the measurements, the spinning rate was set to 10 kHz. Chemical shifts were adjusted by referencing the most deshielded

^{13}C peak of adamantane to 38.5 ppm. Bruker Topspin 3.2 software package was used for data processing.

Single Crystal X-Ray diffraction analysis

X-Ray diffraction patterns of the single crystals of complex **1** were recorded at 298 K using a Bruker D8 Venture diffractometer equipped with an Incoatec ImuS3.0 microfocus sealed-tube MoKα (λ = 0.71073 Å) source and a CCD Photon II detector. The data, collected through generic φ and ω scan, were integrated and reduced using the Bruker AXS V8 Saint Software. The structure was solved and anisotropically refined using the SHELXT and SHELXL packages of the Bruker APEX3 software.[5]

DFT calculations

Amorphous fully hydroxylated SiO_2 slabs[6] (OH density = 7.2 nm^{-2}) with the ancillary ligand bound to the surface of the material were used as initial models for the DFT calculations. Periodic DFT was performed with VASP[7,8] using the PBE exchange-correlation functional,[9] and dispersion corrections at the D3 level.[10] The calculations were carried out using pseudopotentials obtained by the Projected-Augmented Wave (PAW) method[11] as implemented in VASP. The cut-off energy was set to 600 eV. The energy criterion for the convergence of the self-consistent field (SCF) cycles was fixed to 10^{-4} eV. Geometry optimizations were carried out using a conjugate-gradient algorithm and a convergence criterion on the forces exerted on all relaxed atoms of 0.01 eV $Å^{-1}$. After that the Periodic DFT calculations were converged, the cluster models were prepared by truncation of the surface models to the fourth coordination shell of the silicon atom of the heterogenized organic moiety and termination by fluorine atoms. Consequently, a refinement of the cluster edges was performed using the Gaussian 09 software with the pbe1pbe functional and the DEF2svp basis set. NMR calculations were performed within the GIAO framework using ADF[12] 2016 with the PBE0 functional and with the TZP basis set. Tetramethylsilane (for ^{29}Si nuclei) and Nitromethane (for ^{15}N nuclei placed at 380.5 ppm for clarity) calculated at the same level of theory were used as references for chemical shift.

XAS Spectroscopy

XAS measurements were carried out at the Ir L_3 edge at the SuperXAS beamline at SLS (PSI, Villigen, Switzerland). The storage ring was operated at 2.4 GeV in top-up mode with a ring current of around 400 mA. The incident photon beam provided by a 2.9 T super bend magnet source was selected by a liquid nitrogen cooled Si (111) quick-EXAFS monochromator and the rejection of higher harmonics and focusing was achieved by a rhodium-coated collimating mirror for Ir L_3 edge at 2.5 mrad and a rhodium-coated torroidal mirror for Ir L_3 edge at 2.5 mrad. The beamsize on the sample was approximately 200 x 1000 µm. During measurement, the quick XAS monochromator was rotating with 1 Hz frequency in 2 deg angular range and X-ray absorption spectra were collected in transmission mode using ionization chambers specially developed for quick data collection with 1 MHz frequency (gas composition: 2 bar N_2). Spectra were collected for 5 min (300 scans) and averaged. For the Ir L_3 edge, a Zn reference foil was used for energy calibration (9659.0 eV). Samples were diluted with cellulose and pressed as pellets (with optimized thickness for transmission detection).

For the analysis of the X-ray Absorption Near Edge Structure (XANES) the edge energy is defined as the first maximum of the derivative of the XANES region. For the initial processing and normalization, data processing was performed using standard procedures using the ProXASGui software developed at the SuperXAS beamline, PSI, Villigen.[13] The program package Demeter was used for analysis of

EXAFS.[14] S_0^2 value was obtained by fitting Ir(acac)$_3$ reference sample to the crystal structure for the Ir L_3 edge.[15]

Impregnation of 2 with ^{15}N-pyridine

100 mg of the material was exposed to ^{15}N labeled pyridine saturation vapor at room temperature for 10 min. The excess of pyridine was removed under high vacuum (about 10^{-5} mbar) at room temperature for 1 h. The samples were stored under inert atmosphere. Consequently, the ^{15}N CP-MAS NMR spectrum were recorded on a Bruker 600 MHz (14.1 T) spectrometer by using a 3.2 mm HX or HXY probe at 100K.

Formic acid dehydrogenation catalytic experiments

The formation of H_2 and CO_2 in the gas phase was detected with a differential manometer Testo 521–1. Manometric measurements were performed using two homemade jacketed glass reactors coupled to the manometer. In a typical run, the heterogeneous catalyst was weighed with an analytical balance, directly into the measuring reactor and suspended in 4.5 - 4.9 mL of an aqueous solution of HCOOK, taking advantage of the fact that FA dehydrogenation does not take place in alkaline solutions. Both measuring and reference reactors were connected to the manometer and, after equilibration was reached, a suitable amount of pure HCOOH were injected to start the reaction. Catalytic measurements were performed in the presence of air to test the stability of catalysts under conditions useful for possible applications. TOF was derived from the slope of TON vs. t plot in the first minutes of catalysis. TON was evaluated by integrating the residual ^{1}H NMR resonance of HCOOH/K at the end of catalysis, with respect to the initial value. NMR spectra were measured with a Bruker Avance III HD 400 spectrometer equipped with a SmartProbe. Referencing was relative to external TMS (^{1}H and ^{13}C).

Recycling tests were carried out using the general reactions procedure described above. After each run, the catalyst was separated from the reaction mixture by filtering, washed with H_2O (5 mL × 2) and dried under vacuum at 60 °C for 15 hours. The residual solid was then used in subsequent catalytic tests under the same catalytic conditions. The supernatant solution so obtained was thoroughly filtered on celite and then kinetically tested by injecting 70 μL of HCOOH. Ir content in the supernatant solution was quantified by means of ICP-AES analysis. The procedure was repeated for a total of four runs, determining Ir leaching percentage in each run.

CO_2 hydrogenation catalytic experiments

Experiments were performed at the High Throughput Experimentation facility of the ETH Zürich(HTE@ETH) on a custom-made 96-parallel autoclave (ILS GmbH). All the experiments were carried out as follows: a crimped vial equipped with a silicone/PTFE septum containing the catalysts and 5mL of aqueous 1 M base solution was loaded in the batch reactor. Afterwards, the reactor was charged to the desired pressure with a 1:1 H_2/CO_2 gas mixture. The reactor was heated to the desired temperature over 20 minutes and stirred at 500 rpm for the desired reaction time. The reactor was allowed to cool down to room temperature in 1 hour and subsequently vented. Afterwards, 0.5 mL of a 0.1 M aqueous solution of 3-trimethylsilylpropanesulfonate sodium salt were injected in the reaction solution. The resulting mixture was analyzed by means of ^{1}H NMR in D_2O, and the amount of formate was determined by the integration of formate against the internal standard.

To evaluate the effect of the nature of the base on the performance of **Ir_PicaSi_SiO$_2$**, some catalytic experiments were carried out in the presence of triethanolamine (TEA), DABCO and 1,8-

diazabicyclo[5.4.0]undec-7-ene (DBU) (entry 2, Table 2 and entries S1 and S2, Table S7). The determined TON values are the same, within the experimental error, for all three bases as shown in Figure S19.

Synthetic details

Modification of silica beads with PicaSi (PicaSi_SiO$_2$)

In a 500 mL round bottom flask, 5 g of silica beads (60 Å pore size, 70-230 mesh, Silicycle) were suspended in 100 mL of THF. Subsequently, 7 mL of a 2.3M HCl aqueous solution were slowly added to the reaction mixture. The resulting suspension was heated at 70 °C. In the meantime, a homogeneous solution containing 0.6167 g of TEOS (TEOS = TetraEthoxy-Ortho-Silicate) and 0.2834 g of **PicaSi** in 25 mL of THF was prepared. 9 mL of the latter solution were slowly injected in the hot suspension. The resulting mixture were refluxed for 1 h. The solid was collected on a Glass-Fritted Funnel (Porosity 4) and washed with water/THF (20:80, 6x125 mL), ethanol (1x125 mL) and diethyl ether (1x125 mL). The solid was then dried in a glass reactor at 135 °C under high vacuum for 16 hours (Ramp 1 °C min^{-1}) and transferred in a storage tube inside an argon-purged glovebox.

IR (Diffuse Reflectance, □ in cm^{-1}): 3740 (s, OH stretching), 3535 (m, br, OH stretching), 3067 (w, aromatic CH stretching), 2946 (w, aliphatic CH stretching), 2887 (w, aliphatic CH stretching), 1873 (w, br, Si-O stretching overtones), 1670 (m, C=O stretching), 1545 (m, C=C stretching) cm^{-1}. Total surface area: 650 m^2/g; Total pore volume: 1.07 cm^3/g; Mean pore diameter: 6.6 nm. MicroElemental analysis: C = 0.86%, H = 0.34%, N = 0.43%.

^{13}C CP DNP SS-NMR (90%D_2O/10%H_2O, Amupol, δ in ppm): 168, 149, 139, 128, 124, 43, 23, 9. ^{15}N CP DNP SS-NMR (90%D_2O/10%H_2O, Amupol, δ in ppm): 122. ^{29}Si CP DNP SS-NMR (90%D_2O/10%H_2O, Amupol, δ in ppm): -55, -63, -91, -100, -110.

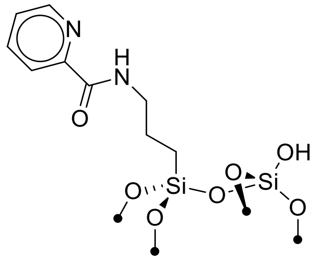

Scheme 1. Proposed Structure of **PicaSi_SiO$_2$**.

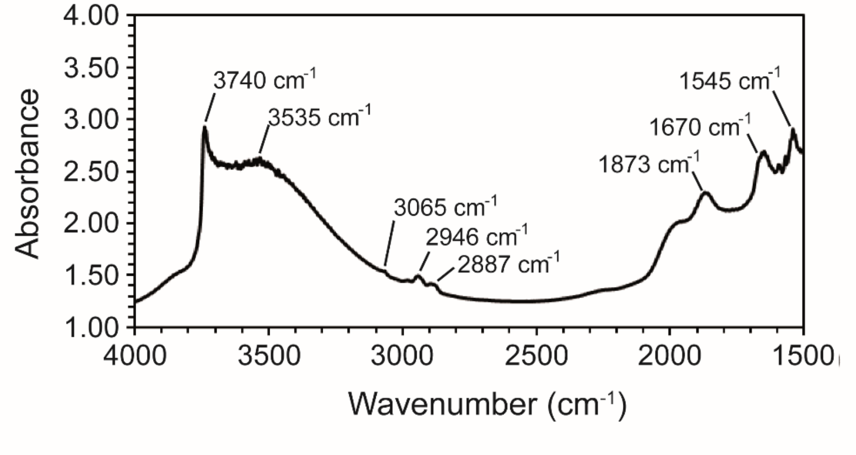

Figure S1. DRIFT-IR spectrum of **PicaSi_SiO$_2$**.

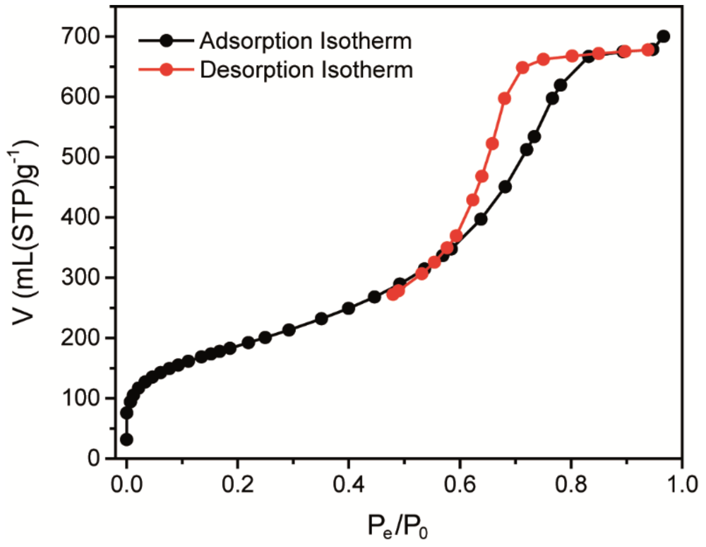

Figure S2. Adsorption and desorption isotherms of **PicaSi_SiO₂**.

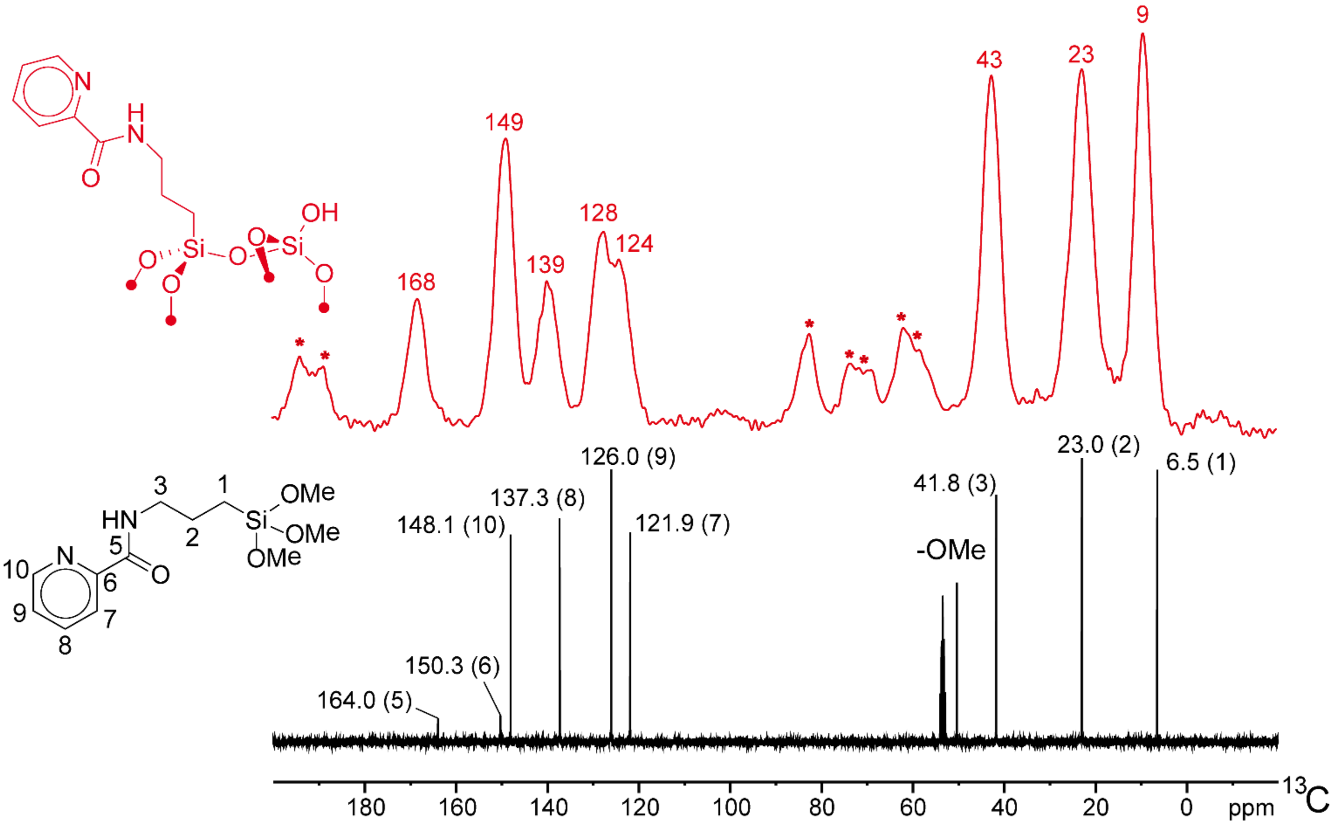

Figure S3. Comparison between the DNP-enhanced ^{13}C CPMAS NMR spectrum of **PicaSi_SiO₂** in red (MAS 10 kHz, 100K, * denotes sidebands) and the $^{13}C\{^1H\}$ NMR of N-(3-(trimethoxysilyl)propyl)picolinamide in black (100 MHz, CD_2Cl_2, 298K).

Immobilization of $[Cp^*IrCl_2]_2$ on $PicaSi\_SiO_2$ ($Ir\_PicaSi\_SiO_2$)

1.5 g of **PicaSi_SiO$_2$** was transferred in a 100 mL Schlenk flask in an argon-purged glovebox. 124.8 mg of $[Cp^*IrCl_2]_2$ (0.65 eq with respect to the ligand) were transferred in a second Schlenk flask and the powder were treated with three cycles of vacuum and argon using a Schlenk line. Subsequently 30 mL of anhydrous dichloromethane were added. The resulting solution was transferred *via cannula transfer* to the Schlenk flask containing **PicaSi_SiO$_2$**. 50 µL of anhydrous triethylamine were added to the reaction mixture and the resulting suspension was stirred for 48 hours at RT. The mixture was filtered with a *cannula* and washed two times with 30 mL of anhydrous dichloromethane. To remove the excess of triethylamine, the solid so obtained was suspended in 50 mL 10^{-4} M H_2SO_4 aqueous solution for 30 minutes. After that, the solid was collected on a Glass-Fritted Funnel (Porosity 4) and washed with water (3x50 mL) and Acetone (3x50 mL). The orange solid was dried for 16 h at 60°C on a high vacuum line (P < 10^{-4} mbar). **Ir_PicaSi_SiO$_2$** was transferred in a storage tube in an argon-purged glovebox.

IR (Diffuse Reflectance, □ in cm^{-1}): 3743 (s, OH stretching), 3390 (s, br, OH stretching), 3075 (w, aromatic CH stretching), 2990 (m, aliphatic CH stretching), 2928 (m, aliphatic CH stretching), 1873 (s, br, Si-O stretching overtones), 1624 (m, C=O stretching), 1595 (m, C=C stretching), 1571 (m, C=C stretching) cm^{-1}. Total surface area: 595 m^2/g; Total pore volume: 0.83 cm^3/g; Mean pore diameter: 5.5 nm. Elemental analysis: C = 2.66%, H = 0.89%, N = 0.32%, Cl = 0.15%, Ir = 2.22%.

^{13}C CP DNP SS-NMR (90%D_2O/10%H_2O, AMUPol, δ in ppm): 173.1, 153.2, 140.8, 127.7, 87.0, 48.4, 23.5, 9.7.^{15}N CP DNP SS-NMR (90%D_2O/10%H_2O, AMUPol, δ in ppm): 56.5.

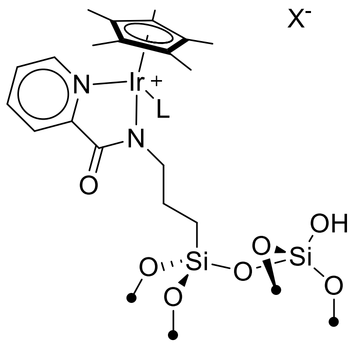

Scheme S2. Proposed Structure of **Ir_PicaSi_SiO$_2$.**

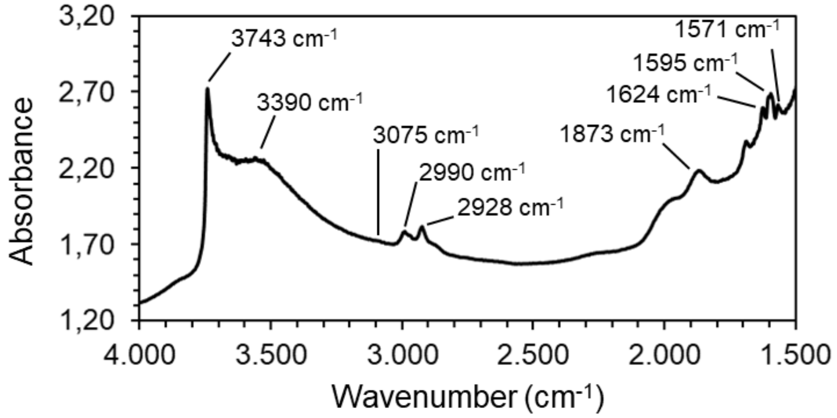

Figure S4. DRIFT-IR spectrum of **Ir_PicaSi_SiO$_2$.**

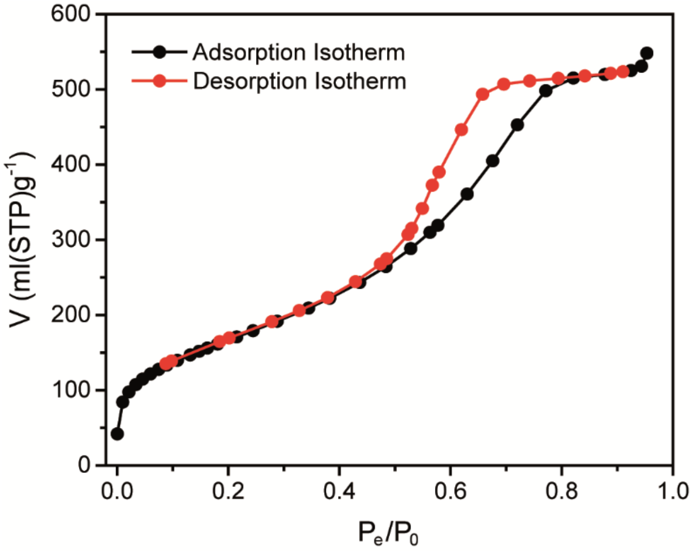

Figure S5. Low-temperature N_2 adsorption/desorption isotherms of **Ir_PicaSi_SiO$_2$.**

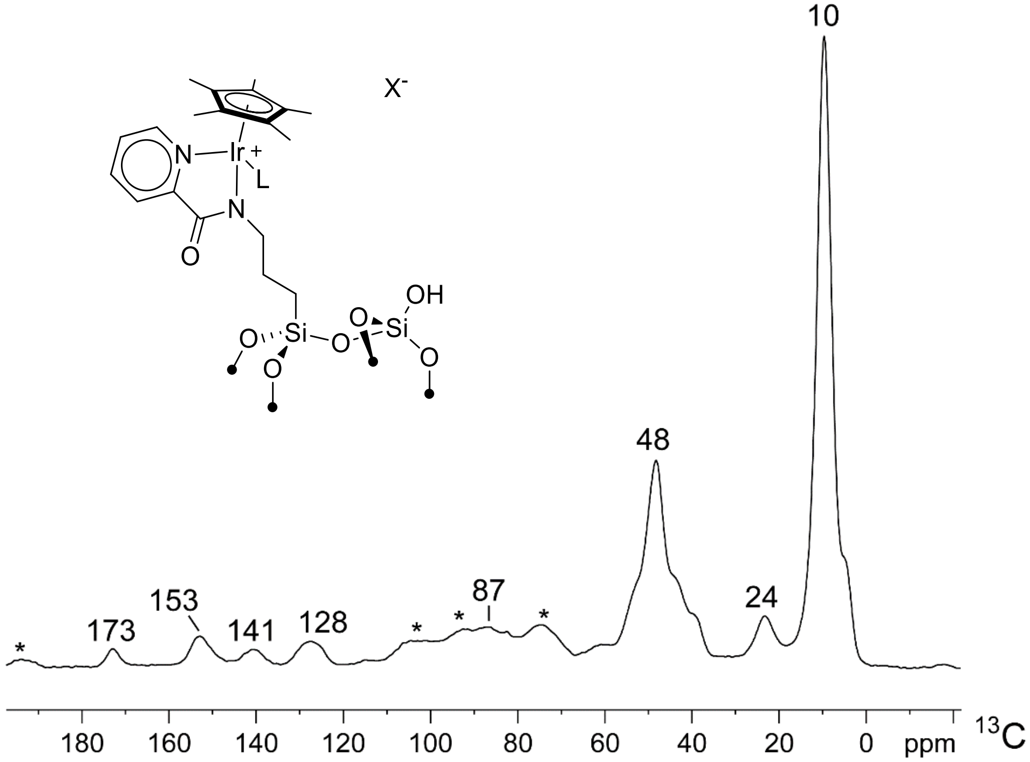

Figure S6. DNP-enhanced ^{13}C CPMAS NMR spectrum of **Ir_PicaSi_SiO$_2$** (MAS 10 kHz, 100K, * denotes sidebands)

Synthesis of (N-propyl)picolinamide

1.0 g of picolinic acid was transferred in a 100 mL 3-necked round bottom flask connected to a Schlenk line. After three vacuum/argon cycles, 5 mL of pure $SOCl_2$ were slowly added to the powder and the so obtained dark suspension was stirred for 4 hours at RT. Subsequently, the solvent was removed under reduced pressure and 30 mL of anhydrous THF were injected in the reactor. The resulting solution was chilled with an ice bath and 1 mL (0.720 g, 1.5 eq.) of anhydrous propylamine; 2.25 mL (1.65 g, 2 eq.) of anhydrous triethylamine were added to the reaction mixture. After 15 min at 273 K, the reaction mixture was refluxed for 24 h. The suspension was allowed to cool down and filtered with a Glass-Fritted Funnel (Porosity 4). The volume of the filtrate was reduced with a rotary evaporator and the resulting dark oil was washed three times with 20 mL of pentane. The residue was then extracted with acetone (10 mL) and dried under reduced pressure. Yield = 1.122 g (84%)

^{1}H NMR (400 MHz, $(CD_3)_2CO$, 298K, δ in ppm, J in Hz): δ = 8.61 (d, $^3J_{HH}$ = 4.5, H10), 8.37 (br, H4), 8.13 (d, $^3J_{HH}$ = 7.8, H7), 7.99 (td, $^3J_{HH}$ = 7.9, $^4J_{HH}$ = 1.7, H8), 7.56 (ddd, $^3J_{HH}$ = 7.7 and 4.7, $^4J_{HH}$ = 1.2, H9), 3.41 (q, $^3J_{HH}$ = 7.0, H3), 1.66 (se, $^3J_{HH}$ = 7.4, H2), 0.97 (t, $^3J_{HH}$ = 7.4, H1).

^{13}C{^{1}H} NMR (100 MHz, $(CD_3)_2CO$, 298K, δ in ppm): δ = 163.7 (s, C5), 150.7 (s, C6), 148.3 (s, C10), 137.5 (s, C8), 126.2 (s, C9), 121.7 (s, C7), 40.7 (s, C3), 22.8 (s, C2), 11.1 (s, C1).

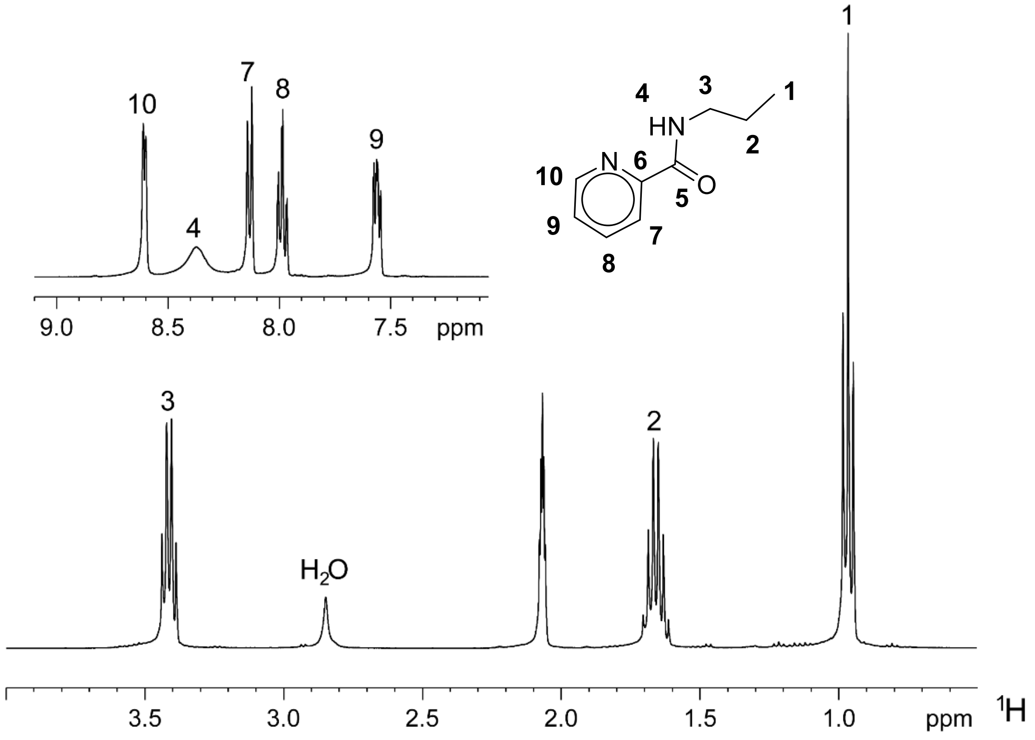

Figure S7. ^{1}H NMR of (N-propyl)picolinamide (400 MHz, $(CD_3)_2CO$, 298K).

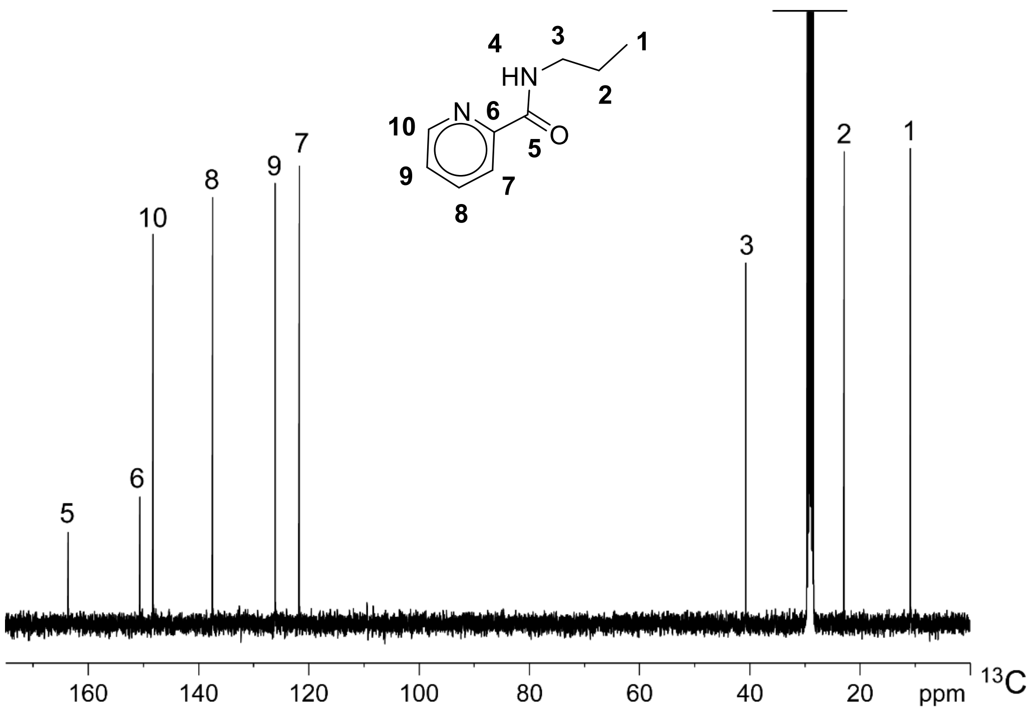

Figure S8. $^{13}C\{^{1}H\}$ NMR of (N-propyl)picolinamide (100 MHz, $(CD_3)_2CO$, 298K).

Synthesis of complex 1

5 mL of a methanolic solution, containing 30.3 mg (2.1 eq., 0.185 mmol) of (N-propyl)picolinamide and 10.3 mg (2.1 eq., 0.185 mmol) of KOH, was slowly added to a suspension of 70 mg (0.14 mmol) of $[Cp^*IrCl_2]_2$ in 10 mL of methanol. The resulting solution was stirred at room temperature for 6 hours, and filtered through filter paper; the solvent was then removed under reduced pressure. The collected solid was contacted with 5 ml of CH_2Cl_2 in order to extract the product. The CH_2Cl_2 solution was filtered and dried through a gentle flux of nitrogen. The product was extracted with 10 ml of diethyl ether and crystallized by slow evaporation of the solvent overnight. Yield = 57.4 mg (59%).

^{1}H NMR (200 MHz, $(CD_3)_2SO$, 298K, δ in ppm, J in Hz): δ = 8.67 (d, $^3J_{HH}$ = 5.7, H10), 8.05 (td, $^3J_{HH}$ = 7.6, $^4J_{HH}$ = 1.7, H8), 7.81 (dd, $^3J_{HH}$ = 8.0, $^4J_{HH}$ = 1.0, H7), 7.64 (ddd, $^3J_{HH}$ = 7.5 and 5.5, $^4J_{HH}$ = 1.6, H9), 4.21 (m, H3a), 3.00 (m, H3b), 1.61 (s, H12), 1.42 (m, H2), 0.84 (t, $^3J_{HH}$ = 7.4, H1).

^{13}C{^{1}H} NMR (50 MHz, $(CD_3)_2SO$, 298K, δ in ppm): δ = 169.9 (s, C5), 154.9 (s, C6), 150.5 (s, C10), 139.1 (s, C8), 127.6 (s, C9), 124.6 (s, C7), 86.2 (s, C11), 50.7 (s, C3), 22.4 (s, C2), 12.0 (s, C1), 8.4 (s, C12).

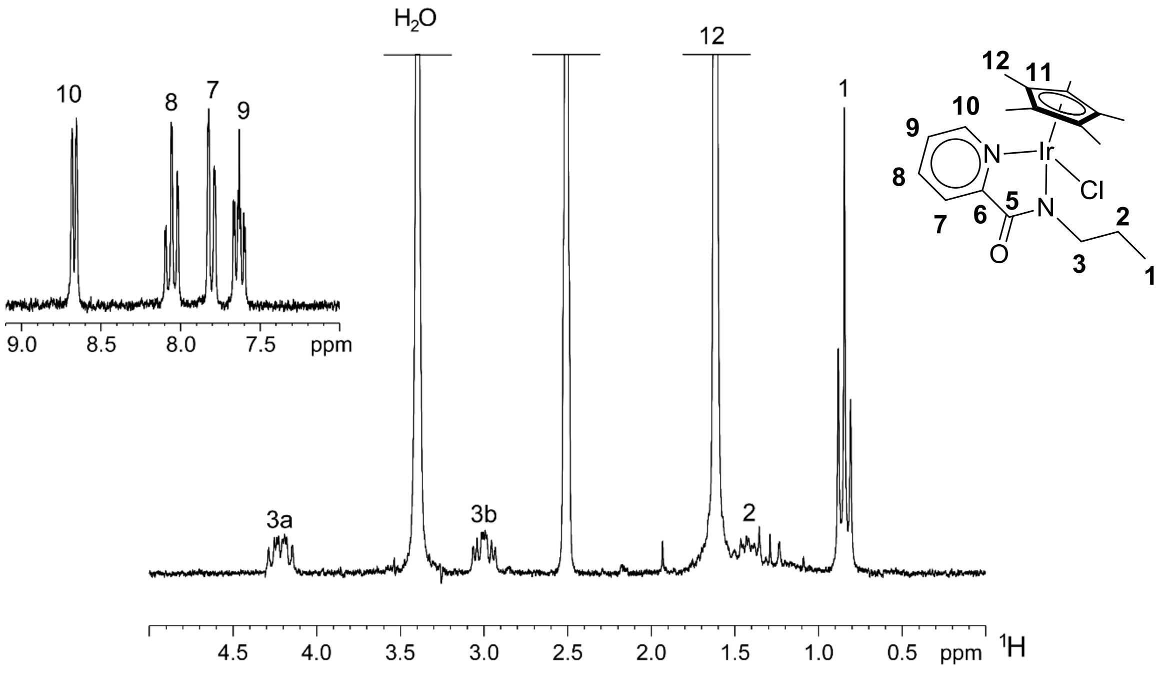

Figure S9. ^{1}H NMR of **1** (200 MHz, $(CD_3)_2SO$, 298K).

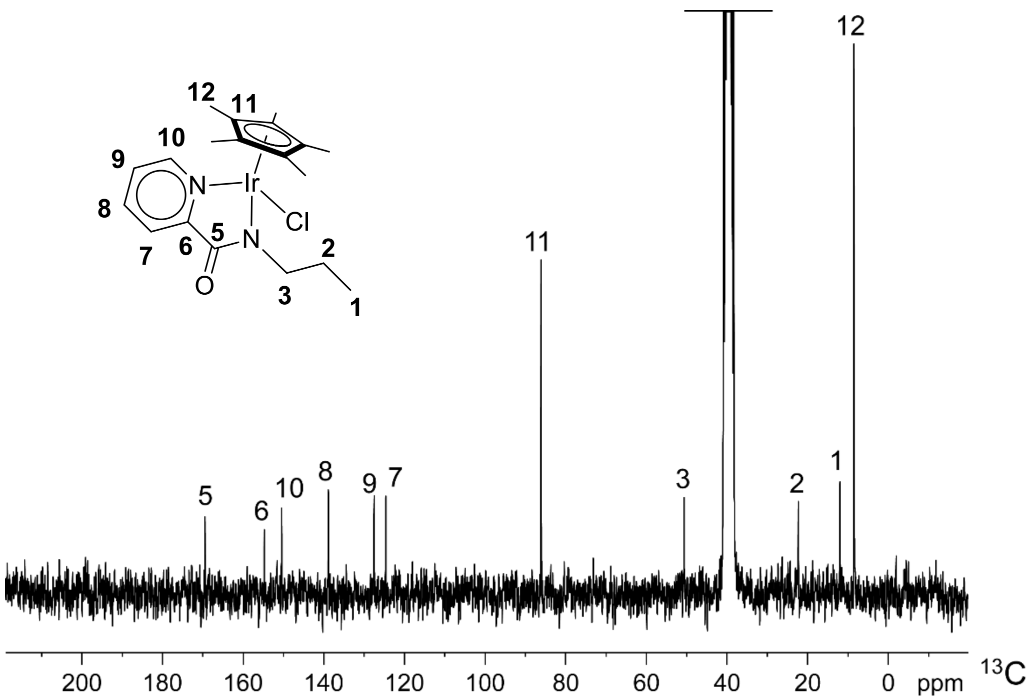

Figure S10. $^{13}C\{^{1}H\}$ NMR of **1** (50 MHz, $(CD_3)_2SO$, 298K).

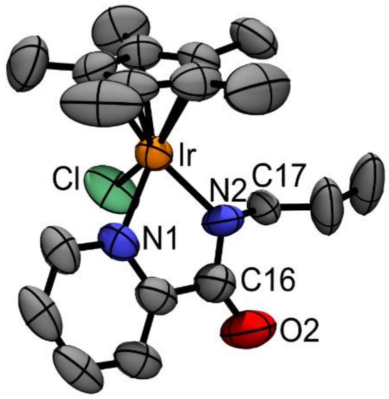

Figure S11. Ortep diagram of complex **1**, ellipsoid at 50% of probability, hydrogen atoms, disorder on the propyl chain and second complex in the asymmetric unit are omitted for clarity. Color code: Ir = orange, N = blue, O = red, C = grey and Cl = aquamarine. Relevant distances (Å) and angles (deg) for **1**: Ir−Cp* = 1.783 and 1.793, Ir−Cl = 2.4107 (17) and 2.404 (2), Ir−N1 = 2.088 (5) and 2.084 (5), Ir−N2 = 2.072 (5) and 2.072 (6), N1−Ir−N2 = 76.1 (2) and 76.1 (2), C16−N2-C17 = 106.4 (18), 120.0 (10), 112.9 (9) and 115.1 (9).

Table S1. Crystal data and structure refinement for complex **1**.

Complex	**1 (CCDC no. 2164448)**
Elemental formula	C19 H26 Cl Ir N2 O
Formula weight	526.07
Crystal system	Orthorhombic
Space group	P 2(1)2(1)2(1)
Unit cell dimensions:	
a = (Å)	8.6757(5)
b = (Å)	15.5492(11)
c = (Å)	29.780(2)
α = (°)	90
β = (°)	90
γ = (°)	90
Volume (Å^3)	4017.3(5)
Z, Calculated density (g/cm^3)	8, 1.740
F(000)	2048.0
Absorption coefficient (mm^{-1})	6.788
Temperature (K)	298(2)
Crystal colour, shape	Orange, Needle
Crystal size (mm)	0.160 x 0.080 x 0.020
On the diffractometer:	
Theta range for data collection	2.4342 to 28.0488°
Limiting indices	-11 ≤ h ≤ 11, -20 ≤ k ≤ 19, -39 ≤ l ≤ 39
Completeness	99.5%
Max. and min. transmission	0.6848 and 0.7457
Reflns collected (not incl. absences)	29031
No of unique reflns, R(int) for equivs	9920, 0.0232
No. of 'observed' reflns (I > 2$\square_I$)	8501
Refinement:	
Data/restraints/parameters	9920/152/500
Goodness-of-fit on F^2	1.035
Final R indices ('obsd' data)	R_1 = 0.0260, wR_2 = 0.0550
Final R indices (all data)	R_1 = 0.0346, wR_2 = 0.0576
Reflns weighted: 1/w = [a]	[\s^2^(Fo^2^)+(0.0222P)^2^]
Largest diff. peak and hole (e. Å^{-3})	0.828 and -0.392

[a] where P = (Fo^2^+2Fc^2^)/3'

DFT ^{29}Si NMR data

Table S2. Isotropic chemical shielding and chemical shift values of the silicon atom of the ancillary ligands calculated from the five models used.

PicaSi_SiO₂ =

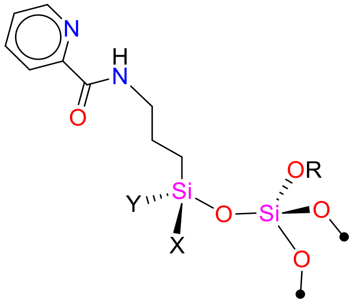

Binding type	R	X	Y	δ_{iso}[a]
	H	OEt	OEt	-44
**T1**	H	OH	OH	-43
	SiF_3	OH	OH	-42
**T2**	H	$OSiF_3$	OH	-56
**T3**	H	$OSiF_2(OH)$	$OSiF_3$	-64

[a] Referenced to TMS.

XAS data

Table S3. Summary of edge energies and white line intensities obtained from analysis of Ir L_3 edge XANES.

**Sample**	**Edge energy /** eV	**White line intensity /** a.u.
**Ir_PicaSi_SiO_2**	11214.75	2.26
**1**	11214.75	2.46
**2**	11214.5	2.80
**$IrCl_3$**	11213.75	2.17
**Na_2IrCl_6**	11213.75	2.28
**$Ir(acac)_3$**	11214.75	2.45
**Ir(COD)(acac)**	11215.0	1.89

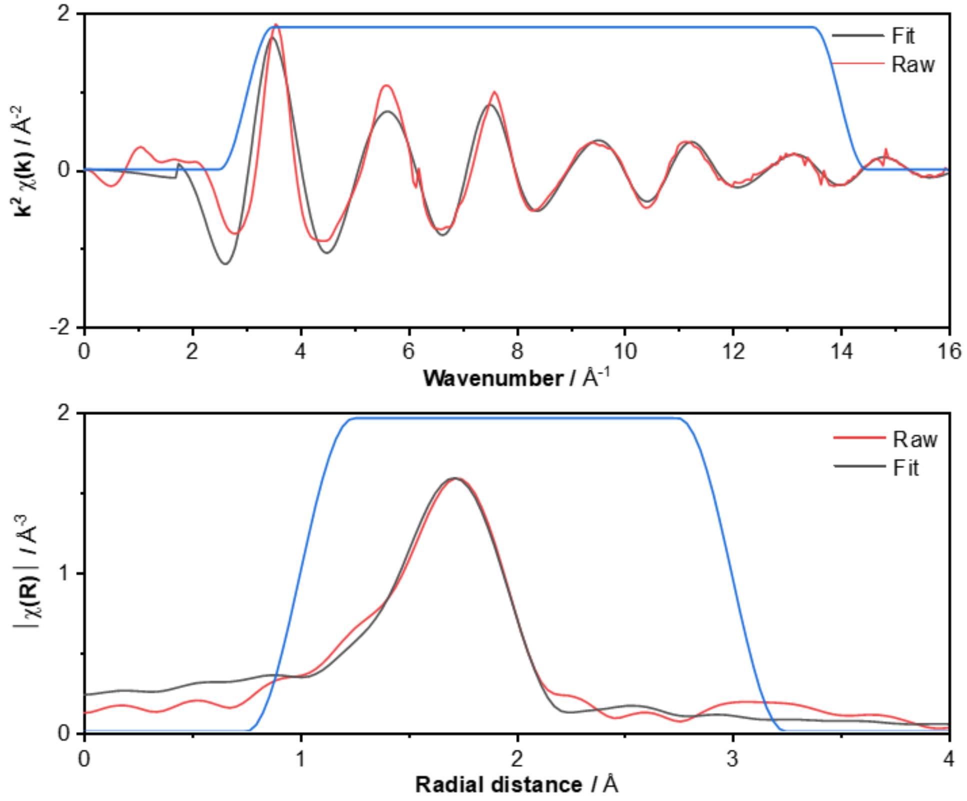

Figure S12. EXAFS fit for $Ir(acac)_3$ (Ir L_3 edge), used to determine amplitude reduction factor (S_0^2). (a) K-space with fit (grey) and raw (red) data. Window (blue) 3.0-14.0 $Å^{-1}$, k-weight = 2, Hanning window, dk = 1; (b) R-space with raw (grey) and fitted (red) data. Window (blue) 1-2.9. Å, k-weight = 2, Hanning window, dR = 0.5. Parameters of fit summarised in **Errore. L'origine riferimento non è stata trovata.**.

Table S4. Summary of fitted parameters for Ir(acac)$_3$ (Ir L_3 edge), 3.0-14.0 Å^{-1}, R = 1-2.9 Å, k-weight = 2. Hanning window, dk = 1Hanning window, dR = 0.5.

Path	Parameter	Value
	E^0 (eV)	11.1 ± 0.8
	S_0^2	0.747 ± 0.050
	σ^2 (Å^2)	0.003 ± 0.001
**Ir-O**	Radial distance (Å)	2.02 ± 0.01
	CN (-)	6 (fixed)
	σ^2 (Å^2)	0.004 ± 0.002
**Ir-C**	Radial distance (Å)	2.91 ± 0.02
	CN (-)	6

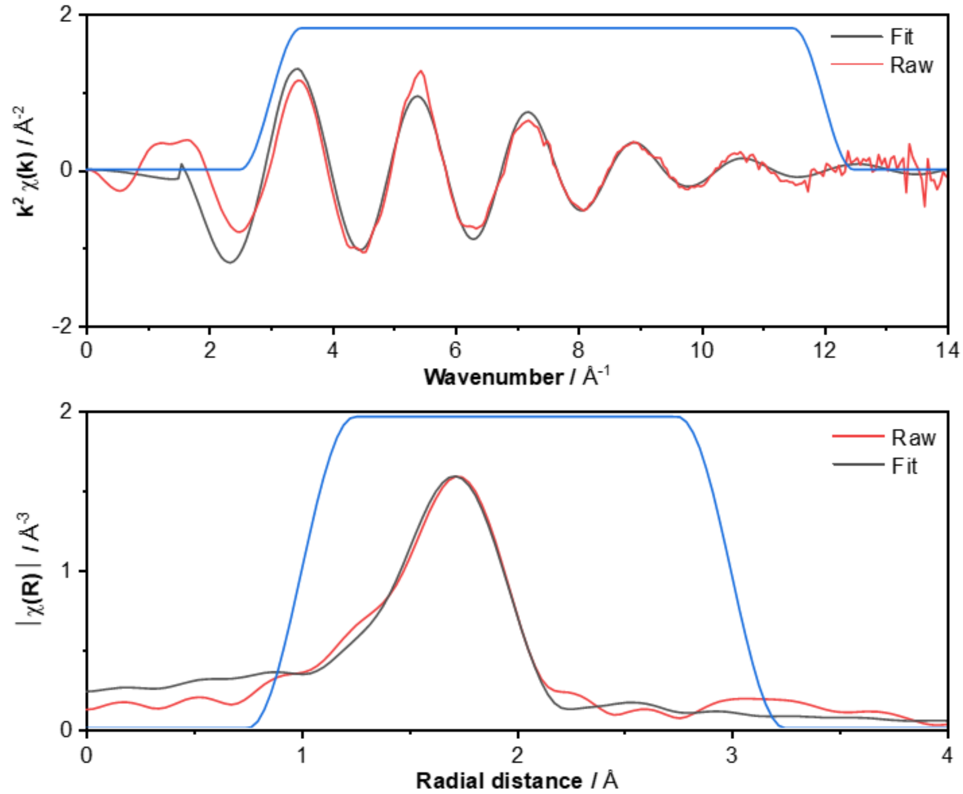

Figure S13. EXAFS fit for **Ir_PicaSi_SiO$_2$** (Ir L_3 edge). (a) K-space with fit (grey) and raw (red) data. Window (blue) 3.0-12.0 Å^{-1}, k-weight = 2, Hanning window, dk = 1; (b) R-space with raw (grey) and fitted (red) data. Window (blue) 1-3. Å, k-weight = 2, Hanning window, dk = 0.5. Parameters of fit summarised in **Errore. L'origine riferimento non è stata trovata.**.

Table S5. Summary of fitted parameters for **Ir_PicaSi_SiO$_2$** (Ir L_3 edge), 3.0-12.0 Å^{-1}, R = 1-3 Å, k-weight = 2. σ^2 and CN for Cp ligands were fixed for all species to reduce the number of variables.

Path	Parameter	Value
	E^0 (eV)	9.0 ± 1.8
	σ^2 (Å^2)	0.004 (fixed)
**Ir-C/N/O**	Radial distance (Å)	2.14 ± 0.04
	CN (-)	5 (fixed)
**Ir-C/N/O**	Radial distance (Å)	2.08 ± 0.03
	CN (-)	3.1 ± 0.8
**Ir-Cl**	Radial distance (Å)	2.34 ± 0.02
	CN (-)	1.2 ± 0.4

^{15}N SS MAS NMR spectrum of impregnated Ir_PicaSi_SiO$_2$ with ^{15}N-pyridine

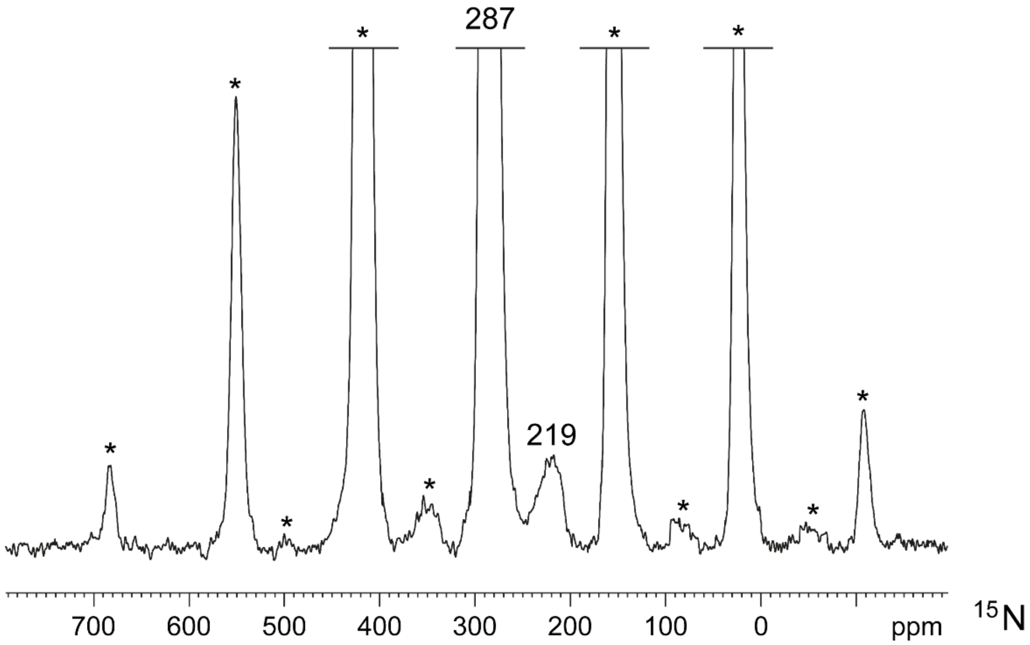

Figure S14. ^{15}N CPMAS NMR spectrum of ^{15}N-pyridine adsorbed over **Ir_PicaSi_SiO$_2$** (MAS 10 kHz, 100K, * denotes sidebands)

Kinetic data of FA dehydrogenation reaction

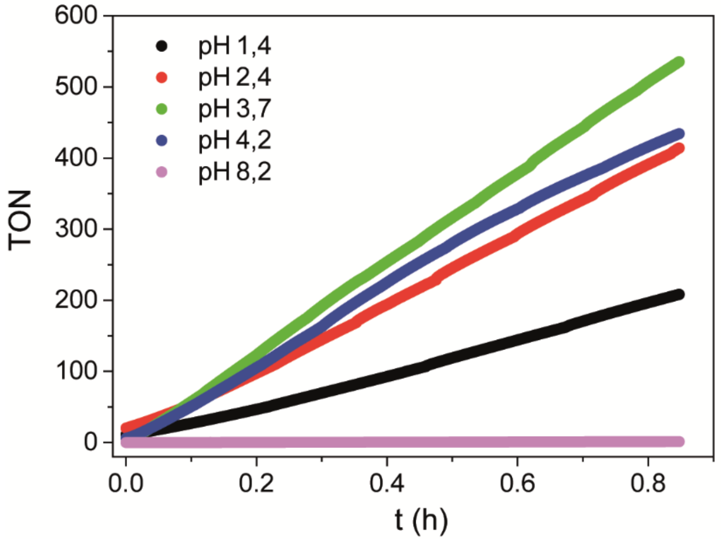

Figure S15. TON vs. t trends for FA dehydrogenation catalyzed by **Ir_PicaSi_SiO$_2$** at different pH ([cat] = 250 μM, ([HCOOH]+[HCOO$^-$]) = 3 M, 298 K).

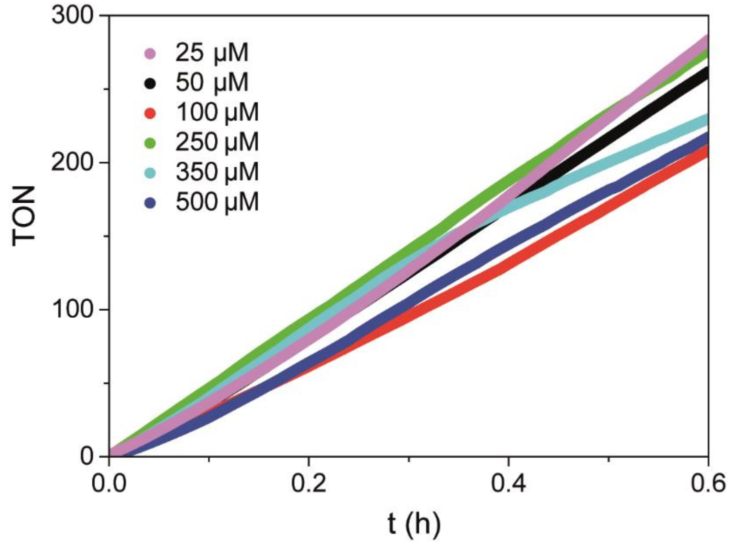

Figure S16. TON vs. t trends for FA dehydrogenation catalyzed by **Ir_PicaSi_SiO$_2$** at different [cat] (([HCOOH]+[HCOO$^-$]) =1 M, pH 3.7, 298 K).

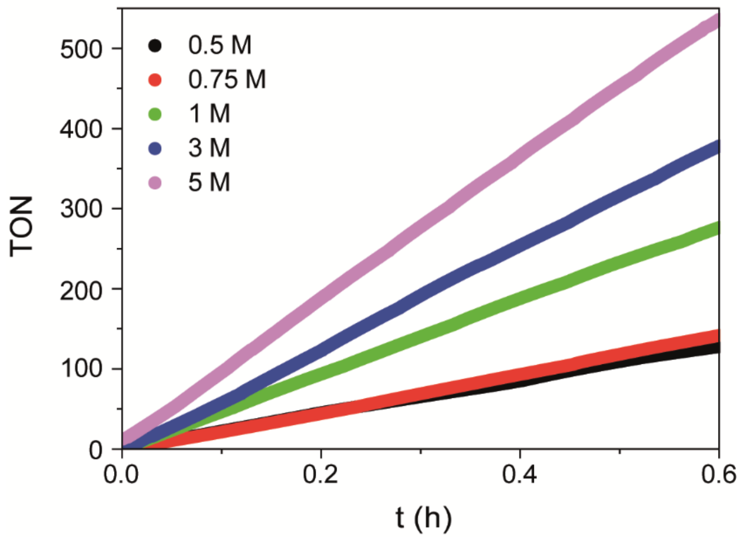

Figure S17. TON vs. t trends for FA dehydrogenation catalyzed by **Ir_PicaSi_SiO$_2$** at different ([HCOOH]+[HCOO$^-$]) ([cat] = 250 μM, pH 3.7, 298 K).

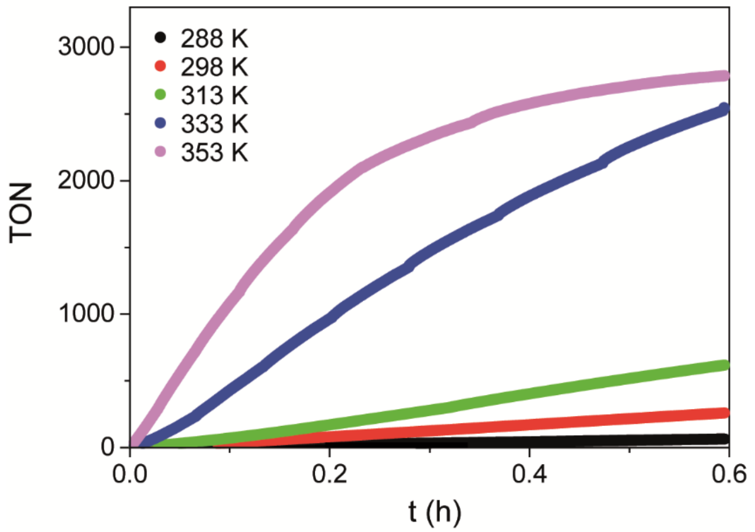

Figure S18. TON vs. t trends for FA dehydrogenation catalyzed by **Ir_PicaSi_SiO$_2$** at different T ([cat] = 50 μM, ([HCOOH]+[HCOO$^-$]) = 1 M, pH 3.7).

Table S6. Catalytic activity of **Ir_PicaSi_SiO$_2$** and the supernatant solution for each run ([HCOOH]+[HCOO$^-$] = 1 M, pH 3.7, 298 K).

	Ir	TOF (h^{-1})	Ir leaching[a]
**Run I**	0.115 μmol/mg	385	5.6 %
**Supernatant I**	0.5 μmol	-	
**Run II**	0.109 μmol/mg	206	4.8 %
**Supernatant II**	0.4 μmol	-	
**Run III**	0.103 μmol/mg	129	3.3 %
**Supernatant III**	0.2 μmol	-	
**Run IV**	0.100 μmol/mg	34	5.3%
**Surpernatant IV**	0.2 μmol	-	

[a] Quantified by ICP-AES.

Kinetic data of CO_2 hydrogenation reaction

Table S7. Effect of the base on the catalytic performances of **Ir_PicaSi_SiO₂** in the CO_2 hydrogenation reaction.

	Cat.	Ir content (μmol)	$n_{Formate}$ (mmol)	[Formate] (M)	Base	t (h)	TON
**S1**	**Ir_PicaSi_SiO$_2$**	0.115	0.58	0.116	TEA	24	5045
**S2**	**Ir_PicaSi_SiO$_2$**	0.115	0.62	0.124	DBU	24	5383

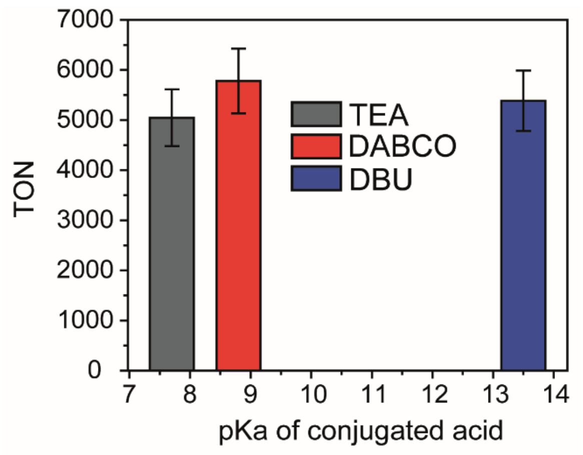

Figure S19. Trend of the TON vs. pKa of the conjugated acid of the base used (t = 24 h, T = 423 K, P = 50 atm with CO_2:H_2 = 1:1 ratio, [Base] = 1M, **Ir_PicaSi_SiO$_2$** content = 0.115 μmol).

References

(1) Ball, R. G.; Graham, W. A. G.; Heinekey, D. M.; Hoyano, J. K.; McMaster, A. D.; Mattson, B. M.; Michel, S. T. Synthesis and Structure of Dicarbonylbis(.Eta.-Pentamethylcyclopentadienyl)Diiridium. *Inorg. Chem.* **1990**, *29* (10), 2023–2025. https://doi.org/10.1021/ic00335a051.

(2) Menendez Rodriguez, G.; Domestici, C.; Bucci, A.; Valentini, M.; Zuccaccia, C.; Macchioni, A. Hydrogen Liberation from Formic Acid Mediated by Efficient Iridium (III) Catalysts Bearing Pyridine-Carboxamide Ligands. *Eur. J. Inorg. Chem.* **2018**, 2247–2250. https://doi.org/10.1002/ejic.201701458.

(3) Rahimi, L.; Mansoori, Y.; Nuri, A.; Esquivel, D. A New Magnetically Retrievable Porous Supported Catalyst for The Suzuki-Miyaura Cross-Coupling Reaction. *ChemistrySelect* **2020**, *5* (37), 11690–11697. https://doi.org/10.1002/slct.202003198.

(4) Begam, H. M.; Choudhury, R.; Behera, A.; Jana, R. Copper-Catalyzed Electrophilic Ortho C(Sp2)-H Amination of Aryl Amines: Dramatic Reactivity of Bicyclic System. *Org. Lett.* **2019**, *21* (12), 4651–4656. https://doi.org/10.1021/acs.orglett.9b01546.

(5) Sheldrick, G. M. Crystal Structure Refinement with SHELXL. *Acta Cryst.* **2015**, *C71*, 3–8. https://doi.org/10.1107/S2053229614024218.

(6) Comas-Vives, A. Amorphous SiO2 Surface Models: Energetics of the Dehydroxylation Process, Strain, Ab Initio Atomistic Thermodynamics and IR Spectroscopic Signatures. *Phys. Chem. Chem. Phys.* **2016**, *18* (10), 7475–7482. https://doi.org/10.1039/c6cp00602g.

(7) Kresse, G.; Furthmüller, J. Efficiency of Ab-Initio Total Energy Calculations for Metals and Semiconductors Using a Plane-Wave Basis Set. *Comput. Mater. Sci.* **1996**, *6* (1), 15–50. https://doi.org/10.1016/0927-0256(96)00008-0.

(8) Kresse, G.; Hafner, J. Ab Initio Molecular-Dynamics Simulation of the Liquid-Metalamorphous-Semiconductor Transition in Germanium. *Phys. Rev. B* **1994**, *49* (20), 14251–14269. https://doi.org/10.1103/PhysRevB.49.14251.

(9) Perdew, J. P.; Burke, K.; Ernzerhof, M. Generalized Gradient Approximation Made Simple. *Phys. Rev. Lett.* **1996**, *77* (18), 3865–3868. https://doi.org/10.1103/PhysRevLett.77.3865.

(10) Grimme, S.; Antony, J.; Ehrlich, S.; Krieg, H. A Consistent and Accurate Ab Initio Parametrization of Density Functional Dispersion Correction (DFT-D) for the 94 Elements H-Pu. *J. Chem. Phys.* **2010**, *132* (15). https://doi.org/10.1063/1.3382344.

(11) Kresse, G.; Joubert, D. From Ultrasoft Pseudopotentials to the Projector Augmented-Wave Method. *Phys. Rev. B* **1999**, *59* (3), 1758–1775. https://doi.org/10.1103/PhysRevB.59.1758.

(12) te Velde, G.; Bickelhaupt, F. M.; Baerends, E. J.; Fonseca Guerra, C.; van Gisbergen, S. J. A.; Snijders, J. G.; Ziegler, T. Chemistry with ADF. *J. Comput. Chem.* **2001**, *22* (9), 931–967. https://doi.org/10.1002/jcc.1056.

(13) Clark, A. H.; Imbao, J.; Frahm, R.; Nachtegaal, M. {\it ProQEXAFS}: A Highly Optimized Parallelized Rapid Processing Software for QEXAFS Data. *J. Synchrotron Radiat.* **2020**, *27* (2), 551–557. https://doi.org/10.1107/S1600577519017053.

(14) Ravel, B.; Newville, M. {\it ATHENA}, {\it ARTEMIS}, {\it HEPHAESTUS}: Data Analysis for X-Ray Absorption Spectroscopy Using {\it IFEFFIT}. *J. Synchrotron Radiat.* **2005**, *12* (4), 537–

541. https://doi.org/10.1107/S0909049505012719.

(15) Isakova, V. G.; Baidina, I. A.; Morozova, N. B.; Igumenov, I. K. γ-Halogenated Iridium(III) Acetylacetonates. *Polyhedron* **2000**, *19* (9), 1097–1103. https://doi.org/https://doi.org/10.1016/S0277-5387(00)00358-2.
